# A Novel Substituted Benzo[*g*]quinoxaline-Based Cyclometalated Ru(II) Complex as a Biocompatible Membrane-Targeted PDT Colon Cancer Stem Cell Agent

**DOI:** 10.1021/acs.jmedchem.4c02357

**Published:** 2024-12-02

**Authors:** Alicia Marco, Jana Kasparkova, Delia Bautista, Hana Kostrhunova, Natalia Cutillas, Lenka Markova, Vojtech Novohradsky, José Ruiz, Viktor Brabec

**Affiliations:** † Departamento de Química Inorgánica, Universidad de Murcia and Murcia BioHealth Research Institute (IMIB-Arrixaca), E-30100 Murcia, Spain; ‡ Czech Academy of Sciences, Institute of Biophysics, Kralovopolska 135, CZ-61 200 Brno, Czech Republic; § Department of Biophysics, Faculty of Science, Palacky University, Slechtitelu 27, CZ-783 71 Olomouc, Czech Republic; ∥ ACTI, Universidad de Murcia, E-30100 Murcia, Spain

## Abstract

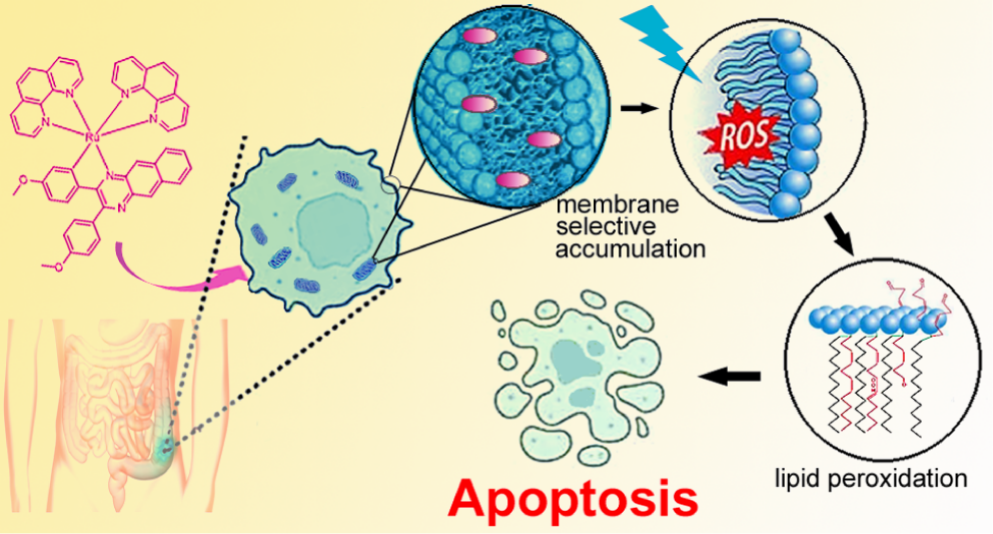

Herein, we describe and investigate biological activity of three octahedral ruthenium(II) complexes of the type [Ru(C^∧^N)(phen)_2_]^+^, **RuL1**–**RuL3**, containing a π-expansive cyclometalating substituted benzo[*g*]quinoxaline ligand (C^∧^N ligand) (phen = 1,10-phenanthroline). Compounds **RuL1**–**RuL3** in cervical, melanoma, and colon human cancer cells exhibit high phototoxicity after irradiation with light (particularly blue), with the phototoxicity index reaching 100 for the complex **RuL2** in most sensitive HCT116 cells. **RuL2** accumulates in the cellular membranes. If irradiated, it induces lipid peroxidation, likely connected with photoinduced ROS generation. Oxidative damage to the fatty acids leads to the attenuation of the membranes, the activation of caspase 3, and the triggering of the apoptotic pathway, thus implementing membrane-localized photodynamic therapy. **RuL2** is the first photoactive ruthenium-based complex capable of killing the hardly treatable colon cancer stem cells, a highly resilient subpopulation within a heterogeneous tumor mass, responsible for tumor recurrence and the metastatic progression of cancer.

## Introduction

Cancer involves a series of sequential and/or simultaneous alterations in molecular pathways that regulate cell proliferation, survival, differentiation, and death.^1^ Despite advances in modern therapeutic modalities, including hormone-based therapy, stem cell therapy, and modern immune checkpoint inhibitors,^2,3^ chemotherapy with cytotoxic small molecule drugs—such as doxorubicin, topotecan, and platinum drugs—remains the cornerstone of cancer management in clinical practice.^4^ However, they are often associated with severe toxicities, suboptimal therapeutic responses, and the emergence of multidrug resistance. Frequently, second tumors occur in many patients after treatment. Therefore, there is an urgent unmet need for novel therapeutic strategies.

**Chart 1:**
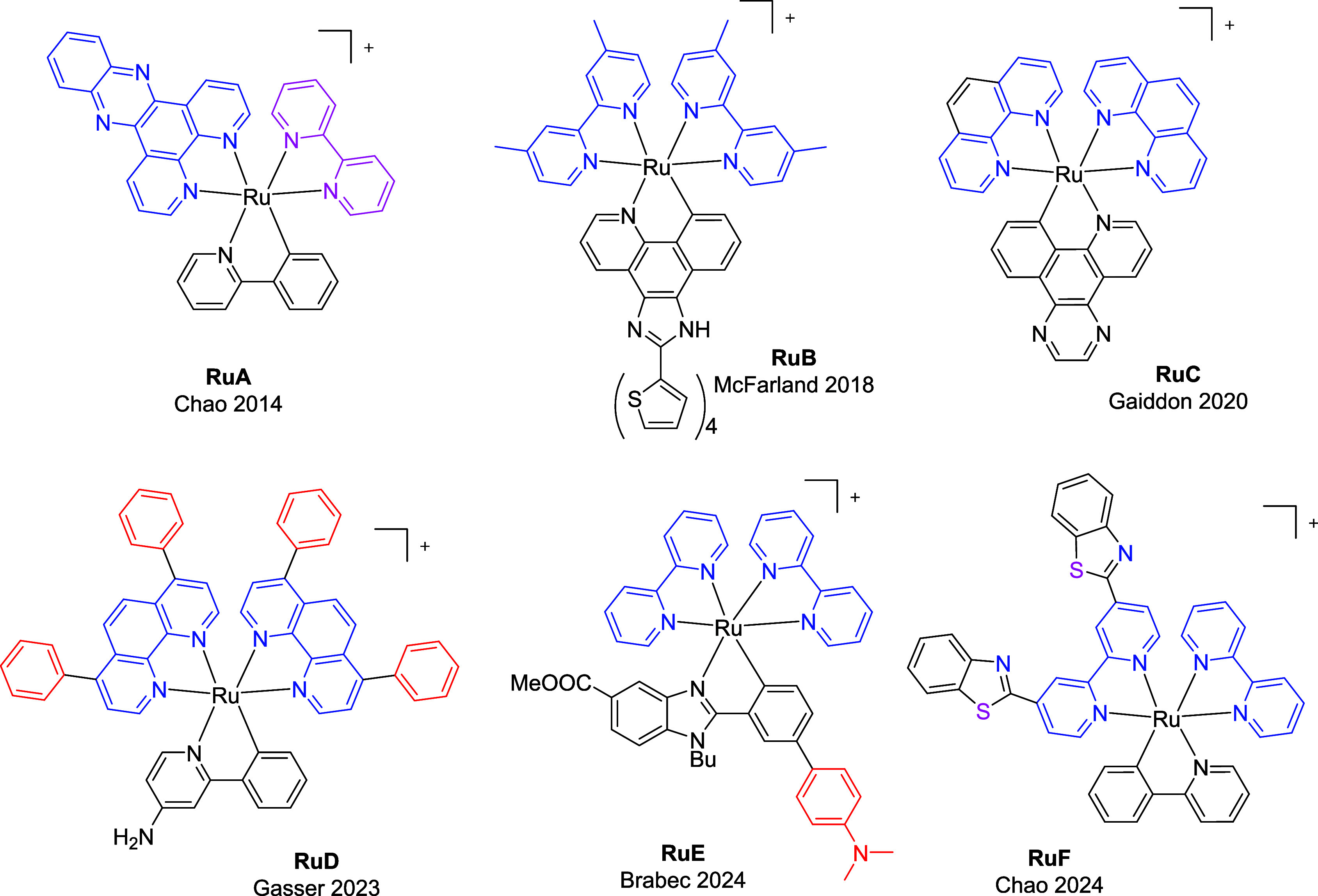
Chemical Structures of Representative Heteroleptic Organo-Ruthenium(II) Complexes Closely Related to This Work

On the other hand, phototherapy offers targeted cancer treatment using light that can be precisely controlled in space and time, reducing the need for invasive procedures.^5^ Transition metal complexes, with their distinctive photophysical and photochemical characteristics, emerge as promising agents for developing new strategies to overcome drug resistance in existing therapies.^6−10^ Photodynamic therapy (PDT) is an approved medical treatment modality with excellent spatiotemporal selectivity and noninvasiveness.^11,12^ The ruthenium(II) complex TLD-1433 has entered phase II clinical trials for the treatment of nonmuscle-invasive bladder cancer using green light.^13,14^ Consequently, Ru complexes have gained significant attention recently for their potential application in PDT.^15−17^ Ru(II) polypyridine complexes surpass the limitations of organic tetrapyrrolic structures thanks to their appealing properties.^18−22^ Cyclometalated Ru(II) compounds have shown promising anticancer properties.^23^ Cyclometalation lowers the energy of the triplet metal-to-ligand charge transfer state (^3^MLCT) and decreases the excited state lifetime of complexes like **RuA** (Chart 1).^24^ Additionally, the anionic nature of the ligand induces a bathochromic shift in the MLCT absorption band of the Ru(II) cyclometalated complex. McFarland et al. investigated the cytotoxic and photocytotoxic activities of a series of ruthenium(II) complexes cyclometalated π-expansive ligands such as **RuB** (Chart 1) and found that the extent of π-conjugation is crucial.^25^ Gaiddon et al. recently reported the light activation of **RuC**, while Gasser and colleagues have described the near-IR absorbing ruthenium(II) photosensitizer **RuD**.^26^ On the other hand, **RuE** triggers oncosis in HeLa cancer cells after irradiation with green light.^27^**RuF** induces synergistic activation of innate and adaptive immunity, leading to oncosis.^28^

Quinoxaline, a nitrogenous heterocyclic compound of interest, is widely used in medicinal chemistry.^29^ In this context, previous studies have investigated the development of quinoxaline-containing ligands that can be utilized as cyclometalating agents for metal ions such as Ir(iii) and Pt(ii) (for example, refs.^30−33^). The electron-deficient quinoxaline ring typically results in longer wavelength absorption and emission features for Ir(III) complexes.^34^ These attributes have led, in particular, to the successful application of these types of complexes to energy upconversion studies^35^ and cellular bioimaging^36^ where efficient longer wavelength absorption is especially advantageous. 2,3-Diphenylbenzo[*g*]quinoxaline (dpbq) ligands are known for their π-expansive properties, making them suitable for PDT applications and other photophysical studies.^37^

**Scheme 1:**
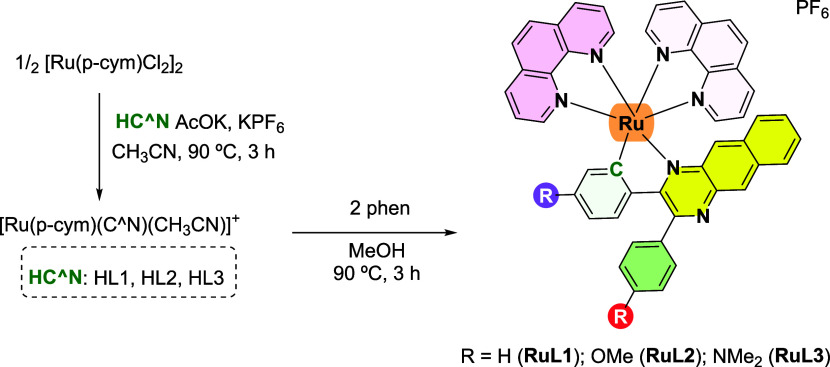
Synthesis of Ruthenium Complexes **RuL1**–**RuL3** Investigated in This Work

Meanwhile, cancer stem cells (CSCs) are a unique subset of cancer cells that play a crucial role in the initiation and maintenance of tumors. They are mainly responsible for driving tumor growth, invasion, metastasis, recurrence, and resistance to chemotherapy.^38−42^ Our groups discovered that octahedral Ir(III) complexes could target malignant CSCs and cause immunogenic cell death (ICD) in melanoma cells.^43^ Recent studies have demonstrated that Ru(II)-based complexes with 2-thiouracil derivatives effectively suppress liver CSCs under dark conditions.^44^ To the best of our knowledge, in this work, we report the first examples of substituted quinoxaline-based cyclometalated ruthenium(II) complexes **RuL1**–**RuL3** (Scheme 1). The C^∧^N ligand was functionalized at either the aryl or quinoxaline units with an electron-donating group (OMe or NMe_2_) as they can shift the absorption of the complexes to the red region.^44^ It is noteworthy that **RuL2** is capable of killing not only the bulk of cancer cells but also the hardly treatable colon CSCs responsible for tumor recurrence and the metastatic progression of cancer and for implementing membrane-targeted PDT.

## Results and Discussion

### Synthesis and Characterization of Ruthenium Complexes (**RuL1**–**RuL3**)

Proligands **HL1**–**HL3** were obtained in good yield following the condensation of 2,3-diaminonaphthalene with the corresponding dione in the presence of trifluoroacetic acid (Scheme S1).^45^ NMR and ESI-MS spectra of the new proligand HL3 are shown in Figures S1, S2, and S10. Dark red/violet solids **RuL1**–**RuL3** (Scheme 1) were obtained in a reaction microwave via two-step synthesis following an optimized procedure adapted from literature.^27^ First, cyclometalation is carried out by a reaction between the ruthenium dimeric precursor, [(η^6^*-p*-cymene)RuCl(μ-Cl)]_2_ and the corresponding proligand HC^∧^N in acetonitrile at 90 °C for 3 h. Then, the corresponding unstable ruthenium intermediate is reacted with phen for 3 h at 90 °C in methanol in a microwave reactor, obtaining the ruthenium cyclometalated complex.

Ruthenium cyclometalated complexes were isolated as hexafluorophosphate salts and purified by column chromatography (2:8, CH_3_CN:DCM) in a 35–40% yield fully characterized by elemental analysis and NMR spectroscopy in CD_3_CN (Figures S3–S8) and mass spectrometry. The purities of the new Ru complexes were higher than 95%, as shown by RP-HPLC/MS (Figure S9) using acetonitrile:water as a mobile phase in gradient mode (Table S1). ESI-MS spectra from HPLC-MS displayed [M – PF_6_]^+^ with the expected isotopic distribution (Figure S11). The ^1^H NMR spectra were recorded in CD_3_CN; in every case, they showed aromatic hydrogen peaks between 9.50 and 6.00 ppm. As expected, two different methyl signals were also observed for both **RuL2** and **RuL3**, corresponding to the MeO or NMe_2_ groups, respectively, of their quinoxaline-based C^∧^N ligands. These signals indicate cyclometalation occurring as expected (note that they are equivalent in the free ligand). The frequency separations in these inequivalent methyl resonances were approximately 0.6 ppm (e.g., δ 3.91 and 3.27 ppm for OMe groups of **RuL2**).

### Crystal Structure by X-ray Diffraction

**Figure 1:**
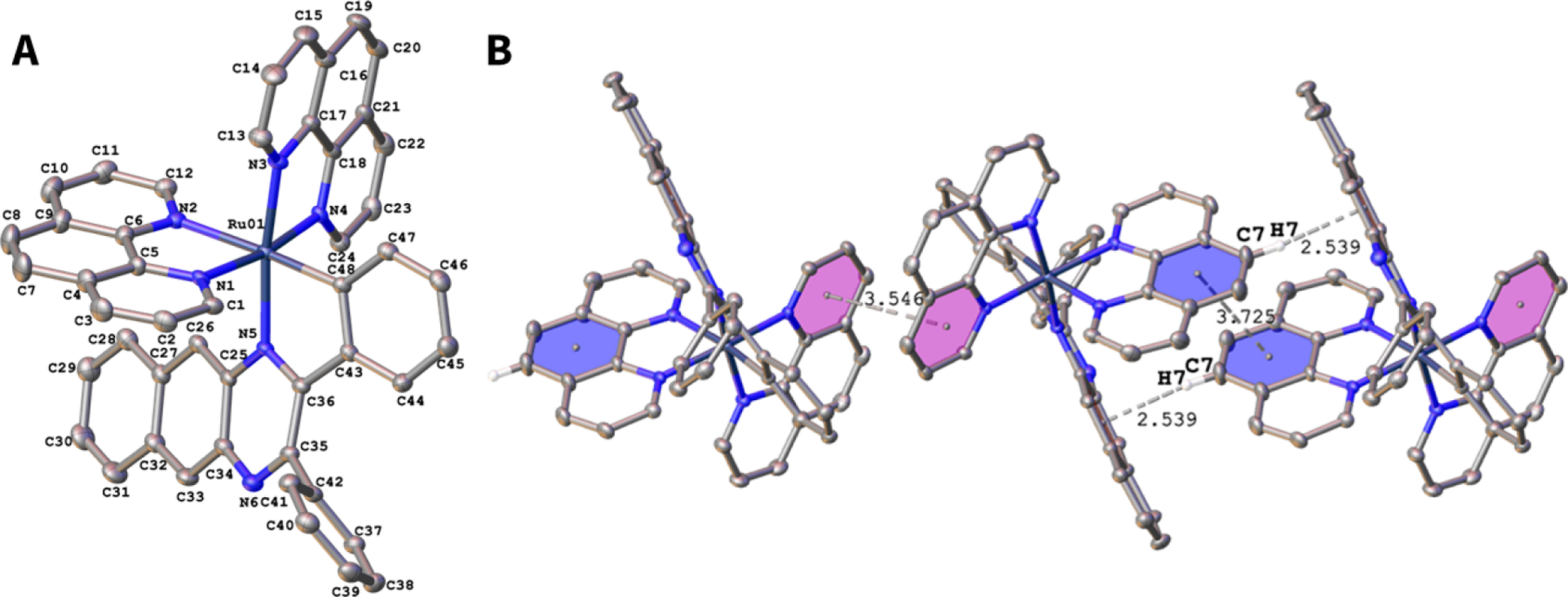
(A) ORTEP plot of the cation of complex **RuL1**. Hydrogen atoms, counterion, and solvent molecules are omitted for clarity. Ellipsoids have been represented at 50% probability. Selected bond lengths (Å) and angles (deg) for **RuL1**: Ru–C48:2.016(3), Ru–N5:2.103(2), Ru–N1:2.068(2), Ru–N2:2.168(2), Ru–N3:2.054(2), Ru–N4:2.050(2). C48–Ru–N5:79.34(9), N1–Ru–N2:78.34(8); N3–Ru–N4:80.01(8). CCDC reference number for **RuL1**: 2387012. (B) π–π and C–H···π interactions in the packing of RuL1 in the crystal, indicated as dashed black lines. Details of these interactions, including the symmetry transformations, are given in Tables S3 and S4 (C_g_ = ring centroid).

The crystal for the X-ray structure of **RuL1** was fortuitously grown through slow solvent evaporation from an NMR tube containing a solution of **RuL1** in CD_3_CN. The single-crystal X-ray determination of **RuL1** confirmed the proposed heteroleptic octahedral structure for the new metal complexes (Figure 1A, Table S2).

Crystallographic data are given in Tables S2-S5. The Ru atom has a distorted octahedral coordination geometry. The Ru–N_phen_ bond distances (2.054–2.168 Å) and Ru–C48 (2.016(3) Å) are within the range reported for ruthenium cyclometalated. The *trans* influence of the σ-bound C donor atom is reflected in a longer Ru–N2 distance of 2.168(2) Å. The π–π interactions between the phen ligands of **RuL1** are shown in Figure 1B, together with C–H···π interactions (see also Figure S13). Additionally, a C–H···F interaction is also observed (Figure S12).

### Photophysical Characterization of the Compounds

**Figure 2:**
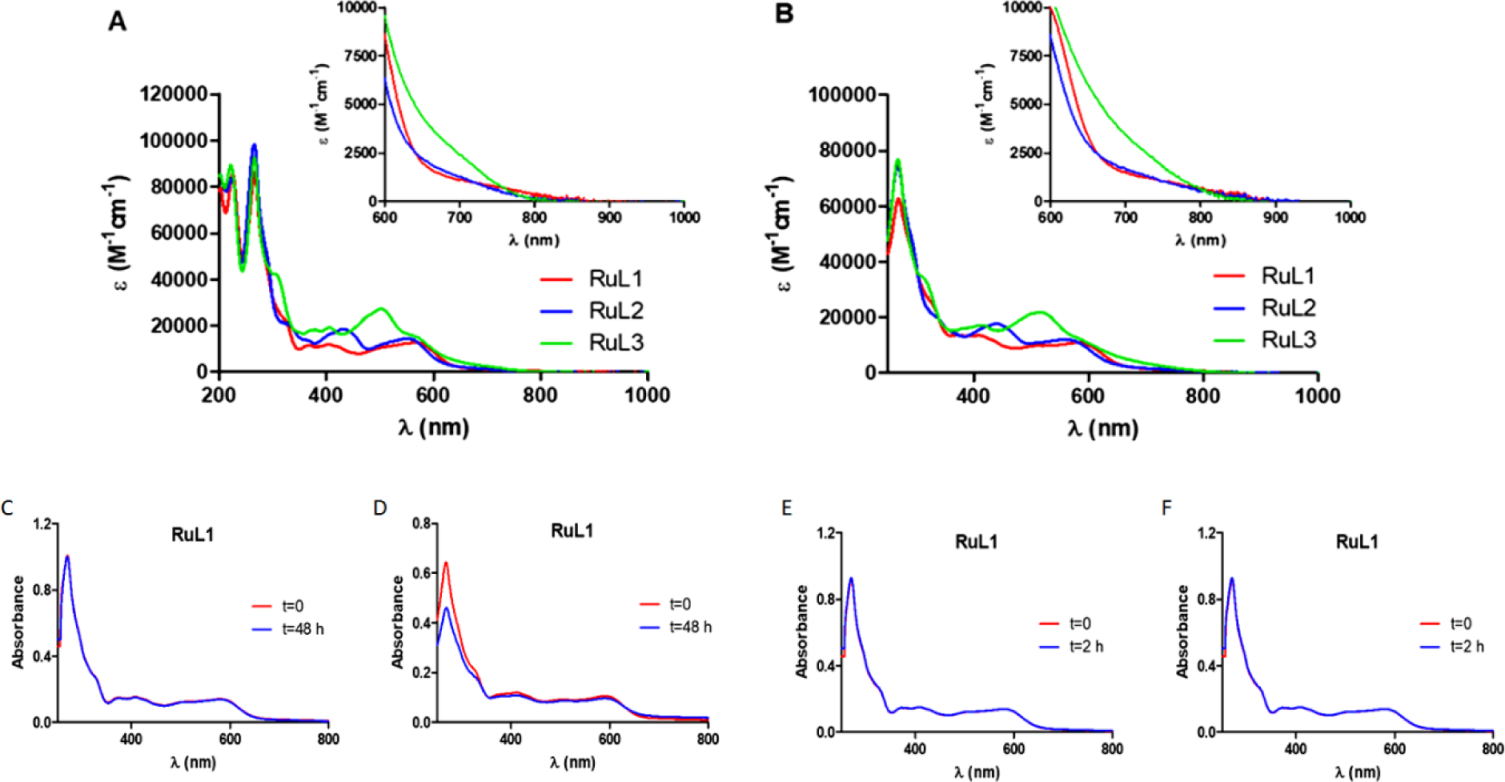
UV/vis absorption spectra of **RuL1**–**RuL3** in (A) acetonitrile and (B) water (1% DMSO) under air at room temperature. Inlet: magnification of the absorption in the red/NIR region. Time evolution of the absorption spectrum of complex **RuL1** (10 μM) in (C) DMSO, (D) RPMI (5% DMSO), (E) DMSO after irradiation with blue light (465 nm, 5 mW cm^–2^), and (F) DMSO after irradiation with red light (620 nm, 15 mW cm^–2^) for 2 h.

The UV/vis absorption spectra of complexes **RuL1**–**RuL3** (10 μM) were recorded in water (1% dimethyl sulfoxide, DMSO) and acetonitrile (Figure 2A,B and Table S6) at room temperature. As can be observed in Figure 2A,B, all UV/vis absorption spectra of the cyclometalated ruthenium complexes show intense sharp bands between 250 and 350 nm that can be assigned to spin-allowed π–π*. These charge transitions are characterized by extinction coefficients around 80000 M^–1^ cm^–1^ in both solvents, the highest values corresponding to complex **RuL3**. **RuL1**–**RuL3** also exhibit small absorption maxima around 560 nm with an absorption tail up to 800 nm. In those complexes containing donor substituents, a bathochromic shift occurs in the lower energy bands, especially in the case of compound **RuL3**, probably due to the strong electron-donating ability of the dimethylamino group. It should be noted that the observed molar absorption coefficient values in the visible region are suitable for red light-driven applications.

### Stability and Photostability Studies of **RuL1**–**RuL3**

The dark and light stabilities are essential for good photosensitizers. For that reason, the stabilities of complexes **RuL1**–**RuL3** in the dark were studied for 48 h by UV/vis spectroscopy, both in DMSO and in RPMI cell culture medium (5% DMSO) at 37 °C (Figure 2C,D for **RuL1** and Figures S14 and S15 for **RuL2** and **RuL3**). No changes were observed. Subsequently, the photostability of the metal complexes was studied under constant irradiation in DMSO using blue light (465 nm, 5 mW cm^–2^) and red light (620 nm, 15 mW cm^–2^). As shown in Figure 2E,F (for **RuL1**) and Figure S16 (for **RuL2** and **RuL3**), their absorption spectra remained unchanged after light exposure for 2 h.

### Evaluation for ^1^O_2_ and/or •OH Photogeneration in Cell-Free Media

**Figure 3:**
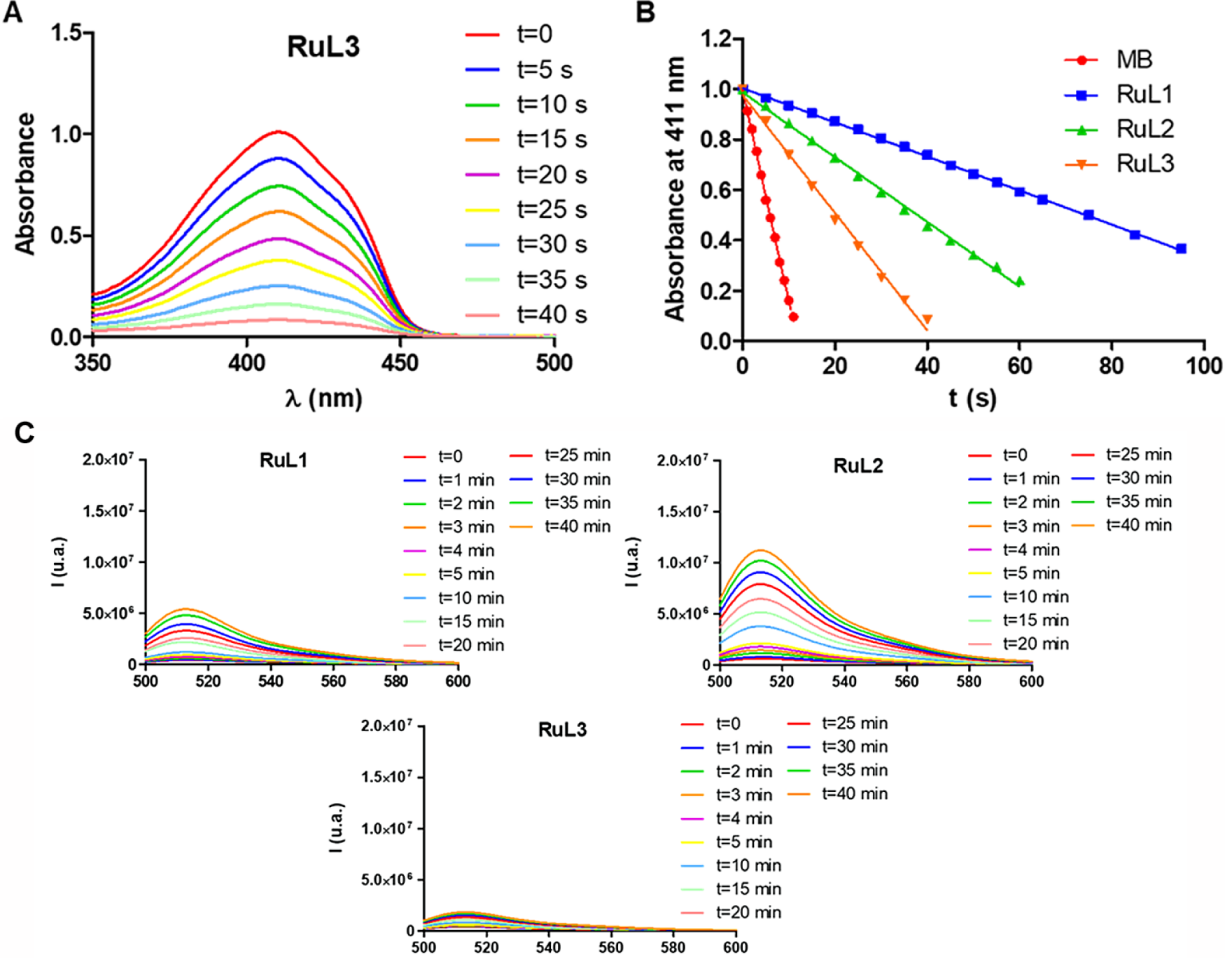
(A) Decrease in the absorption intensity of DPBF in the presence of **RuL3** after irradiation with red light (620 nm, 5.06 mW cm^–2^) in acetonitrile. (B) Representation of absorbance at 411 nm vs irradiation time of the DPBF solution in the presence of **RuL1**–**RuL3** with red light (620 nm, 5.06 mW cm^–2^). Methylene blue was used as a reference. (C) Increase of the fluorescence spectra emission of 3′-*p*-(hydroxyphenyl)fluorescein (HPF) (10 μM) upon photoirradiation of complexes **RuL1**–**RuL3** (10 μM) with red light (620 nm, 20 mW cm^–2^). HPF fluorescence was excited at 490 nm.

We investigated the ability of the new ruthenium(II) complexes to generate singlet oxygen (^1^O_2_) through an energy transfer (type II PDT) process. The ability to photocatalytically convert molecular oxygen into singlet oxygen was evaluated by UV/vis spectroscopy in acetonitrile using 1,3-diphenylisobenzofuran (DPBF) as a singlet oxygen probe. In the presence of ^1^O_2_, the absorption intensity of DPBF at 411 nm decreases. The UV/vis spectra of DPBF in the presence of the different complexes, **RuL1**–**RuL3** (3.5–7 μM), were monitored at different times of red light irradiation (620 nm, 5.06 mW cm^–2^) (Figure 3A for **RuL3** and Figure S17 for **RuL1**–**RuL2**). In order to obtain singlet oxygen quantum yields, methylene blue was used as a reference. As observed in Figure 3B (and Table S7), the new ruthenium complexes exhibited a low singlet oxygen quantum yield, with **RuL3** being the major producer (∼17%).

We also investigated if the new complexes can produce hydroxyl radicals (OH•) by electron transfer (type I PDT) photoreactions following red light irradiation. This could overcome the diminished therapeutic effect in tumor hypoxic microenvironments. The spectroscopic method is based on oxidizing the nonfluorescent 3′-*p*-(hydroxyphenyl)fluorescein (HPF) to the corresponding fluorescent product in the presence OH•. Thus, if OH• is formed, the emission intensity at 514 nm increases when excitating at 490 nm.^46^ The emission spectra of the solutions containing the compound (10 μM) and HPF (10 μM) in PBS (5% DMF) were recorded at different times after the irradiation with red light (620 nm, 20 mW cm^–2^).

As shown in Figure 3C, under red light irradiation, both **RuL1** and **RuL2** increased the fluorescence intensity of HPF, whereas **RuL3** did not produce hydroxyl radicals in tested conditions. We can conclude that the substituent plays an important role in the generation of ROS (both singlet oxygen and hydroxyl radical) in cell-free media.

### Phototoxic Activity on Cancer Cells

The dark- and photoactivities of the complexes have been tested on a panel of three human cancer cell lines of different origin, namely, cervical adenocarcinoma HeLa, melanoma A375, and colon carcinoma HCT116 cells. These lines were selected because the tumor tissues from which they originate are readily accessible for PDT treatment. The cells seeded in a 96-well plate were treated with Ru complexes diluted in EBSS for 1 h in the dark and subsequently irradiated for 1 h with blue (420 nm, 58 W m^–2^) or red (613 nm, 20 W m^–2^) light. After the irradiation, the Ru-containing EBSS was removed, and cells were allowed to recover for a further 70 h. The number of live cells was determined using a standard MTT assay.

The resulting data are summarized in Table 1. Although all investigated complexes **RuL1**–**RuL3** showed activity at the micromolar scale in the dark, their activity was further potentiated by irradiation with blue or red light; blue light was significantly more effective in this respect than the red one, with phototoxicity indexes (PTIs) reaching values 17–100. Among the tested compounds, **RuL1** was the least sensitive to irradiation, demonstrating the lowest PTI in all cell lines. In contrast, the other two, **RuL2** and **RuL3**, were markedly more susceptible to photopotentiation; the PTI values determined for those complexes after blue light irradiation ranged in higher tens and even reached a value of 100 for the **RuL2** complex in the HCT116 cell line, indicating an excellent intensification of biological activity due to irradiation.

**Table 1:** IC_50_ values (μM)^a^ Obtained for Cancer Cells Treated with the Investigated Ru Complexes in the Dark or after Irradiation as Determined by the MTT Assay^b^

	Dark	Blue	PTI_Blue_	Red	PTI_Red_
HeLa					
**RuL1**	2.0 ± 0.3	0.12 ± 0.02	16.6	0.27 ± 0.07	7.4
**RuL2**	1.9 ± 0.4	0.06 ± 0.01	31.6	0.13 ± 0.03	14.6
**RuL3**	2.5 ± 0.2	0.033 ± 0.006	75.8	0.14 ± 0.01	19.2
A375					
**RuL1**	1.3 ± 0.2	0.05 ± 0.01	26.0	0.11 ± 0.02	11.8
**RuL2**	1.2 ± 0.2	0.023 ± 0.003	52.2	0.07 ± 0.02	17.1
**RuL3**	1.3 ± 0.2	0.022 ± 0.004	59.0	0.06 ± 0.02	21.7
HCT116					
**RuL1**	0.9 ± 0.2	0.045 ± 0.005	20	0.15 ± 0.02	6
**RuL2**	0.8 ± 0.1	0.008 ± 0.002	100	0.08 ± 0.01	10
**RuL3**	1.08 ± 0.09	0.013 ± 0.001	83.1	0.06 ± 0.01	18

a Data represent mean ± SD from at least 3 independent experiments, each performed in triplicate.

b PTI (Phototoxicity index) was calculated using the following formula: PTI_(Blue,Red)_ = IC_50_ (dark-nonirradiated cells)/IC_50_ (irradiated cells; Blue, Red).

Although the Ru-complexes show some selectivity for tumor versus noncancerous cells even in the dark (Table 2), their photopotentiation may still represent a considerable benefit. During photodynamic chemotherapy, tumor tissue is irradiated selectively so that the substantial photoenhancement at the site of the tumor can significantly elevate the difference between the effects on cancer (irradiated) and healthy (nonirradiated) tissue.

**Table 2:** IC_50_ Values (μM)^a^ Obtained for Noncancerous MRC5pd30 Cells Treated with the Investigated Ru Complexes in the Dark as Determined by the MTT Assay

	MRC5pd30	SI^b^
**RuL1**	12 ± 4	8.6
**RuL2**	7 ± 1	5.4
**RuL3**	9.3 ± 0.9	5.7

a Data represent mean ± SD from 3 independent experiments performed in triplicate.

b SI (selectivity index) was calculated using the following formula: SI= IC_50_ (MRC5pd30, dark)/average IC_50_ (cancer cells, dark).

Many Ru complexes have been described in the literature as damaging mitochondria and affecting their physiological functions.^47,48^ So, the results obtained by the MTT assay (which is based on mitochondrial metabolization of MTT) could potentially be affected by the effect of Ru complexes on mitochondrial metabolism. Therefore, the results of phototoxicity experiments have also been verified for the most sensitive HCT116 cells with the use of SRB (Sulforhodamine B) assay. The test is based on measuring cellular protein content, i.e., it reflects the number of living cells independently on mitochondrial metabolism. As indicated (Table S8), the SRB assay fully confirmed the results found by MTT, with IC_50_ in good agreement for both MTT and SRB assays (the IC_50_ values differ within the range of experimental errors). The data indicate that Ru complexes tested in this work do not affect mitochondrial dehydrogenases involved in reducing MTT.

### Cellular Accumulation

Ru complexes investigated in this work were prepared with the intention of studying their (photo)activity against tumor cells. An important precondition for the biological action of metallopharmaceuticals is their ability to penetrate and accumulate in cells. Therefore, the accumulation of Ru in HCT116 cells (the most sensitive cell line with the highest PTI) was quantified by inductively coupled plasma mass spectrometry (ICP-MS). After 2 h of incubation of HCT116 cells with Ru compounds in the dark, the amount of Ru associated with cells treated with **RuL1**, **RuL2**, and **RuL3** was 527 ± 11, 504 ± 26, and 503 ± 33 ng Ru/10^6^ cells, respectively, which roughly correlates with dark activities in this cell line. As all three complexes do not differ substantially in both intracellular accumulation and antiproliferative activity in the dark (no significant differences proven by *t* test), the prominent differences in activities after irradiation (Table 1) may, therefore, be likely related to the different photophysical properties of the individual complexes.

### ROS Induction in Cells

**RuL1**-**RuL3** have been shown to induce ROS production when irradiated in cell-free media (see above–photogeneration of ^1^O_2_ and/or •OH). ROS can induce oxidative stress at their high nonphysiological concentrations in cells, leading to cell damage and death. So, increased phototoxicities of these complexes after irradiation could be attributed to their ability to arouse reactive oxygen species (ROS). To confirm this view and determine whether the complexes can induce ROS in living cells, the CellROX assay was employed. CellRox green reagent is cell-permeant and aims to detect and quantify ROS in live cells. To address possible quantitative differences between the individual complexes, HCT116 cells were treated with **RuL1**–**RuL3** at their equimolar (45 nM) concentration for 1 h, irradiated with blue light (or kept in the dark) for 1 h and, immediately after irradiation, amount of intracellular ROS was determined by Flow Cytometry.

In the cells treated in the dark, the level of ROS remained comparable to control, untreated cells (Figure S18, full columns), indicating that antiproliferative activity in dark conditions is likely unrelated to the elevated ROS levels. Noticeably, irradiation of cells pretreated with the Ru complexes resulted in a significant increase in intracellular ROS concentration (Figure S18, empty columns); a correlation can be observed between the ability to induce intracellular ROS in the irradiated cells and the phototoxicity of **RuL1**–**RuL3**. This points to a significant contribution of ROS and subsequent oxidative stress to the striking enhancement of the biological activity of Ru complexes in irradiated cells.

Further experiments aimed to understand the mechanism of photoactivity and elucidate the cellular responses to the action of the studied complexes under irradiation. The **RuL2** complex was selected as a model compound because, if irradiated with blue light, it showed both the highest phototoxicity and the highest PTI in the most sensitive HCT116 cell line.

### Modality of Cell Death

**Figure 4:**
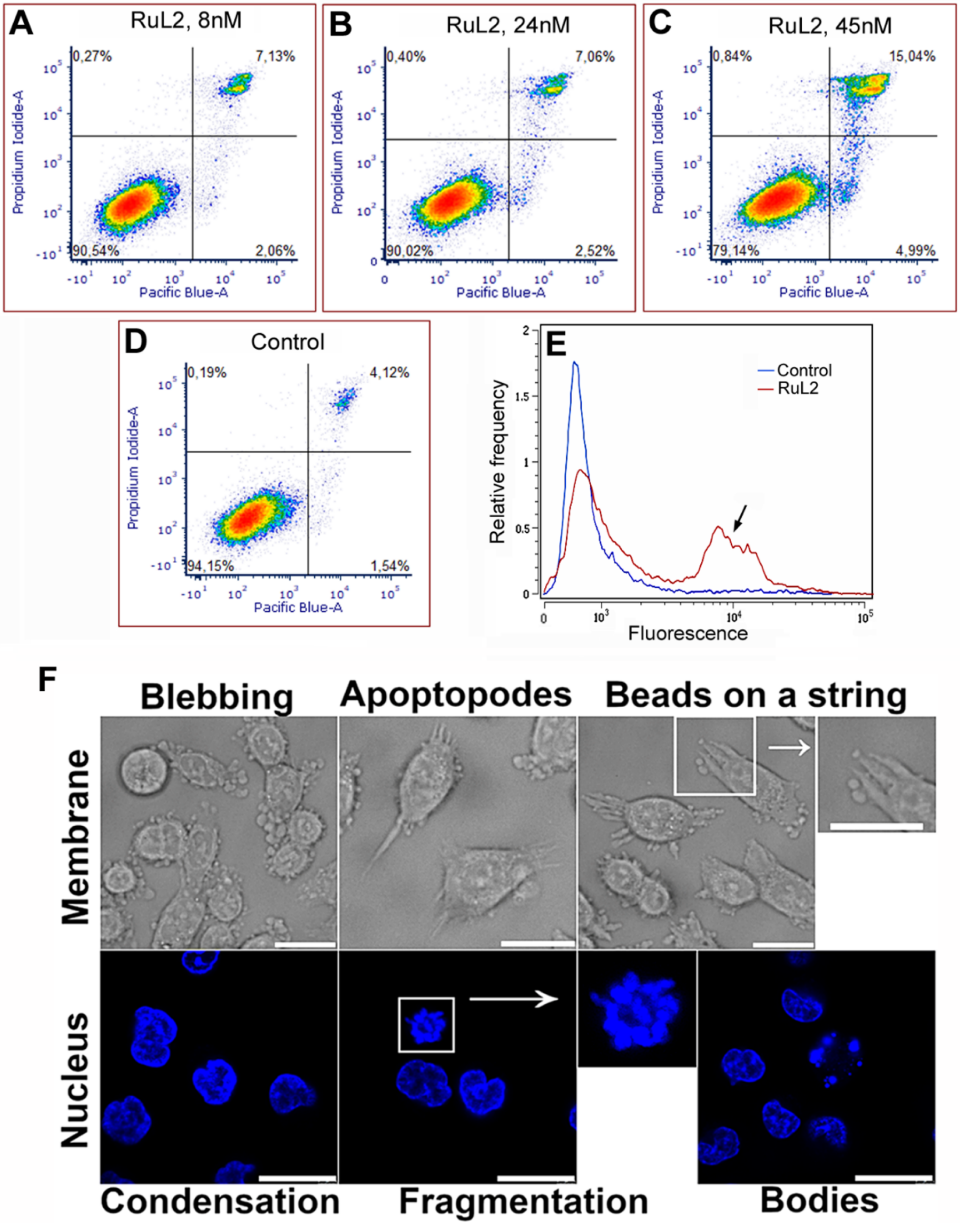
(A–D) Representative density plots of HCT116 cells after their PI/annexin V Pacific blue staining. Before staining, cells were incubated with **RuL2** in the dark (1 h) and irradiated with blue light (1 h). Control, untreated cells were irradiated as well. Then, the cells were allowed to recover in Ru-free media for 24 h. Early apoptotic cells are in the right lower quadrant (annexin V-positive, PI-negative), whereas cells undergoing necrotic processes are in the left upper quadrant (annexin V-negative and PI-positive). The signals in the right upper quadrant (both annexin V and PI-positive) represent dead (necrotic and late apoptotic) cells. (E) Activation of caspase 3 in HCT116 cells as detected by CellEventCaspase3/7 Green Detection Reagent using flow-cytometry. Representative histograms of untreated irradiated control (in blue) or cells treated and irradiated with **RuL2** are shown. Caspase 3 positive cell population is indicated by arrow; 10,000 cells were analyzed in each sample. (F) Apoptotic morphological features assessed in HCT116 cells following treatment with **RuL2**. Cells were exposed to 50 nM of **RuL2** for 2 h (1 h in dark, 1 h irradiation with 420 nm light). Morphological alterations in the cytoplasmic membrane were evaluated using bright field microscopy 90 min postirradiation. The scale bar in the bright field images represents 20 μm. Apoptosis-related changes in nuclear morphology were analyzed through confocal microscopy utilizing Hoechst dye staining. The time-lapse observations of nuclear morphological changes included nuclear condensation and fragmentation at 90 min and the formation of apoptotic bodies at 150 min after irradiation. Scale bars in the confocal images denote 20 μm.

To reveal the mode of cell death, a dual annexin V and propidium iodide (PI) staining assay was employed. Panels A–D in Figure 4 show the effect of irradiated **RuL2** on HCT116 cells. A significant elevation of the number of cells undergoing apoptosis (PI^–^/annexin^+^ cells, right bottom quadrant) was apparent after 24 h of post-treatment incubation (panels A–C in Figure 4) as compared to the control, untreated irradiated cells (panel D in Figure 4). Simultaneously, almost no increase of necrotic cells was detectable (PI^+^/annexin^–^, left upper quadrants). This may suggest an apoptotic pathway as a predominant mechanism of cell death.

However, annexin V/PI dual staining is not unequivocally conclusive to confirm apoptosis because annexin positivity with simultaneous impermeability for PI has also been described for cell death modes other than apoptosis.^49,50^ Therefore, to verify the apoptotic mode of cell death, activation of caspase 3 was also tested. This enzyme is responsible for proteolysis during apoptosis, and detecting cleaved caspase 3 is therefore considered a reliable marker for cells dying by apoptotic pathways.^51^ As indicated (panel E in Figure 4), a noticeable increase in the caspase 3 activity was observed 24 h after the cells were incubated and irradiated with the **RuL2** complex. Thus, combined with the annexin V/PI staining results, the result suggests a caspase-dependent apoptosis as the predominant mode of cell death.

### Morphology of the Cells

The study of morphology-related features is a critical area of research in understanding cell death mechanisms. Apoptosis is characterized by a series of biochemical and morphological changes; the morphological alterations are critical indicators of apoptosis and are often utilized in assessing cell death mechanisms. Morphological features of apoptosis, such as cell shrinkage, membrane blebbing, chromatin condensation, and the formation of apoptotic bodies, can be effectively visualized using various microscopy techniques, including bright field and confocal microscopy.^52^ These techniques can, therefore, be used for verification apoptosis in HCT116 in response to the treatment with **RuL2** after irradiation with blue light. Detailed microscopic analysis (panel F in Figure 4) allowed us to correlate morphological changes with biochemical markers of cell death.

The morphological changes observed in the cytoplasmic membrane (panel F in Figure 4, top images) are consistent with the apoptosis process, which is often characterized by particular alterations in cellular membrane-specific morphology, including cell shrinkage (pyknosis), membrane blebbing (strictly related to casp-3 activation), formation of apoptopodes (PANX1 activation) and formation of beads on string structures.^53^ The nuclear changes typically observed during the apoptotic process encompass chromatin condensation, followed by nuclear fragmentation, a phenomenon known as karyorrhexis, which ultimately leads to the formation of apoptotic bodies.^54,55^

To assess nuclear morphology changes, cells were stained with Hoechst dye, and time-lapse confocal microscopy (0–150 min) was chosen to point out critical phases in the apoptotic process, such as chromatin condensation and fragmentation, which are indicative of apoptosis. Chromatin condensation represents an early event in apoptosis, where the chromatin becomes densely packed, reflecting the cell’s transition toward programmed death. This is succeeded by karyorrhexis, where the nuclear envelope disintegrates, and the nuclear material is fragmented into smaller pieces. The culmination of these processes results in the formation of apoptotic bodies, which are membrane-bound vesicles containing cellular debris and fragmented nuclear material (panel F in Figure 4, bottom images).

In summary, HCT116 cells showed apoptotic morphology after the treatment with **RuL2** combined with blue light irradiation, confirming apoptosis as a significant form of cell death induced by **RuL2** under blue light irradiation. This finding emphasizes the importance of apoptosis in the context of cellular responses to **RuL2**.

### Subcellular Distribution

Drug distribution and localization within a cell are essential factors in its effectiveness. A drug needs to enter the cells and reach the intracellular compartment that houses its target for its action to manifest.^56^ Therefore, knowing the drug’s distribution in the cell could be profitable for determining the mechanism of action.

For this purpose, fractionation of HCT116 cells treated by the **RuL2** was performed using a FractionPREP Cell Fractionation Kit, and the amount of Ru associated with each fraction was determined by ICP-MS. The vast majority of Ru (95 ± 3%) was associated with a membrane/particulate fraction, which is likely related to the high lipophilicity of the complex. Only a negligible portion of Ru was found in the nucleus (nuclear proteins and membrane), cytosol, and cytoskeleton-containing fractions (ca. 1–2% in each fraction). The results thus indicate that the phototoxic effect of **RuL2** can be mainly caused by damage to cell membranes, either plasma membranes or membranes of intracellular organelles.

### Imaging of Membranes in Living Cells

**Figure 5:**
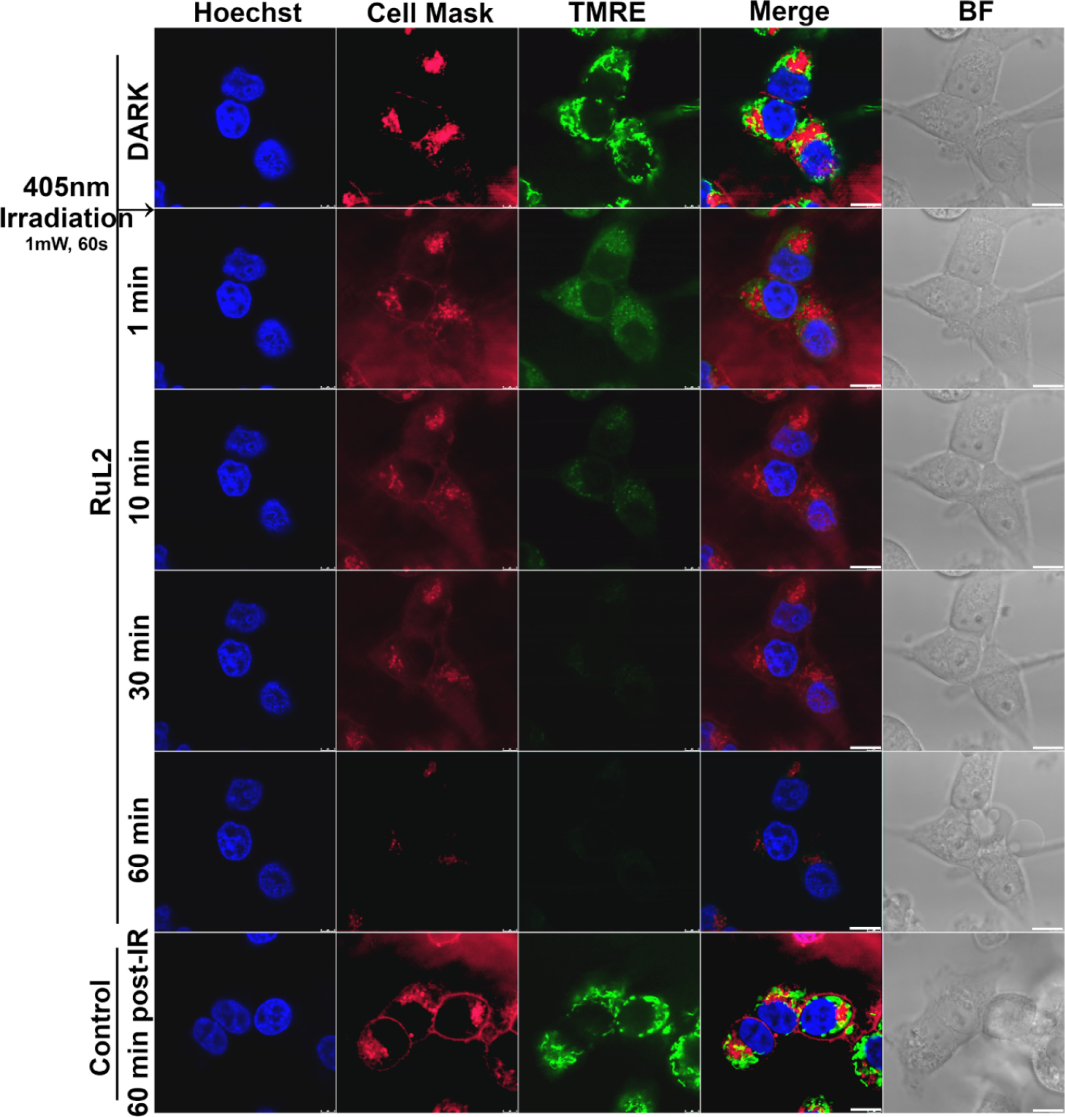
Analysis of HCT116 cells by confocal microscopy. Cells were treated with 80 nM of **RuL2** for 2 h in EBSS. Then, samples were stained with Hoechst 33342, CellMask deep red (λ_em_ = 680 nm), and TMRE (λ_em_ = 590 nm, shown in green for clarity). Samples were irradiated with blue laser light 405 nm (1 mW, 60 s). The scale bar in the images represents 10 μm.

For a closer examination of the effect of **RuL2** on the membranes, we used confocal microscopy. The plasma membrane of the HCT116 cells treated with **RuL2** was stained by CellMask Plasma Membrane Stain deep red, while mitochondrial membranes (as a representative of intracellular organelles) were stained by tetramethylrhodamine ethyl ester (TMRE). The nuclei of cells were stained by Hoechst (blue fluorescence) for better orientation in the sample images. The samples were then mounted to a confocal microscope, irradiated with a blue laser (405 nm) for 60 s, and images were taken at the time intervals indicated in Figure 5.

Shortly after the irradiation (1 min), a significant lessening of the signal for both plasma and mitochondrial membranes was observed, accompanied by blurring the fluorescence signal even into areas outside the cells. This decrease proceeded with increasing time so that after 60 min after irradiation, almost no membrane-associated fluorescence was detectable.

CellMask plasma membrane stain is an amphipathic molecule containing a negatively charged hydrophilic fluorescent dye and a lipophilic moiety for anchoring the probe in the plasma membrane. This lipophilic part is embedded between the membrane phospholipids using hydrophobic interactions. Therefore, its release from the membrane may indicate damage to the plasma membrane and disruption of interactions between the phospholipid components of the plasma membrane. Similarly, TMRE is a cell-permeant, cationic fluorescent dye that accumulates rapidly and reversibly in the mitochondria of living cells due to the negative mitochondrial membrane potential. A decrease in fluorescence of TMRE-stained mitochondria indicates a depolarization of the mitochondrial membrane, which can be attributed to the disturbance of this membrane. Thus, the results of this experiment suggest that **RuL2**, accumulated in membranes of cells and intracellular organelles, can cause damage to these membrane structures after irradiation with blue light.

The decrease in fluorescence results from the effect of **RuL2** (and not simply from photobleaching), as evidenced by the fact that the intensities of the signals from the membranes remain unchanged even 60 min after irradiation of control, untreated cells (Figure 5, bottom panels).

### Oxidative Damage to Lipids

The results mentioned above have shown that the **RuL2** complex damages cell membranes. This begs the question of how this damage occurs on a molecular level. Since ROS (reactive oxygen species) are generated after irradiating **RuL2**, it can be assumed that oxidative damage to membrane components, including membrane lipids, may occur. To verify this hypothesis, we monitored oxidative damage to lipids in the membrane of HCT116 cells using sensor dye Bodipy 665/676.^57,58^

**Figure 6:**
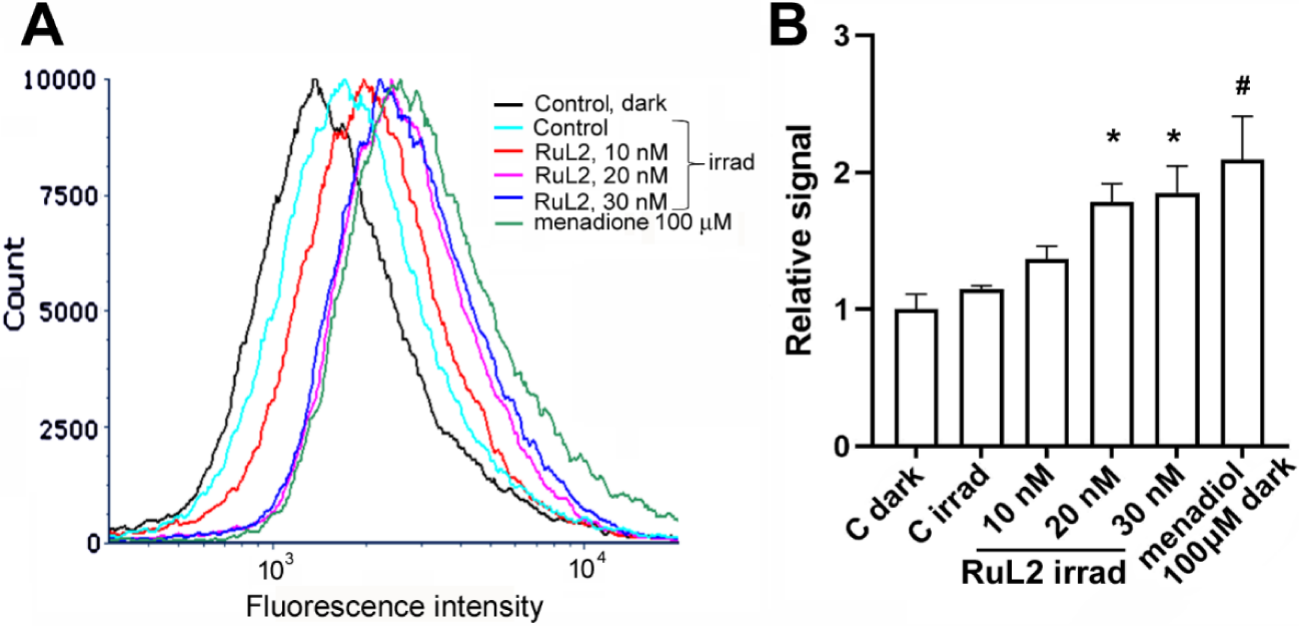
(A) Representative histograms of lipid peroxidation in HCT116 cells analyzed by flow cytometry. Cells were treated for 2 h (1 h dark, 1 h irradiation 420 nm) with an increasing concentration of **RuL2**. Positive control menadiol (100 μM, 2 h in dark) was also included in the experiment. Samples were stained with Bodipy 665/676 lipid peroxidation sensor. (B) Quantitative evaluation of the experiment. Data represent a mean ± SD from two measurements; (3–4) × 10^4^ cells were analyzed in each sample. * = significantly different from irradiated control (*p* < 0.05); # = significantly different from control kept in the dark (*p* < 0.05*)*.

The resulting data indicate a significant fluorescence increase after the cells were treated and irradiated with increasing concentration of **RuL2** (Figure 6); the effect was quantitatively similar to that induced by three thousand times higher concentration of menadiol, an effective agent causing lipid peroxidation in cells.^59,60^ Thus, the analysis confirmed that the production of ROS by the **RuL2** complex localized in cell membranes can ultimately lead to oxidative damage to membrane components, which can subsequently be reflected in cellular processes, including cell death.

It has been shown that oxidative damage to the cell membrane, including ROS-induced lipid peroxidation, plays a crucial role in initiating apoptosis.^61−63^ The integrity of the cell membrane is vital for cell survival, and any compromise, primarily through oxidative stress, can trigger apoptotic pathways. The membrane instability due to the peroxidation of lipids is likely associated with the formation of cleaved fatty-acyl chains.^64^ Lipid peroxidation thus destabilizes the membrane structure, which can disrupt membrane-bound proteins and ion gradients, releasing pro-apoptotic factors such as cytochrome C from mitochondria. This release triggers a cascade of molecular events, activating caspase 3 – the enzyme responsible for executing apoptosis.^65,66^ Taken together, the mechanism of action of **RuL2** can be, based on our results, summarized that due to the redox properties of the Ru-complex, the level of ROS increases owing to the irradiation in the location of the complex, i.e., in the membranes, which causes oxidative damage to membrane lipids and subsequently triggers apoptotic cell death through caspase 3 activation.

### Activity toward Cancer Stem Cells

The experiments described above revealed that the photoactivity of the Ru complexes tested here is closely related to their ability to produce ROS and thus damage cellular membranes. Interestingly, CSCs, a subpopulation of cells within tumors with the ability to self-renew and drive tumorigenesis, exhibit a unique response to ROS. Studies have shown that CSCs often maintain lower ROS levels than bulk, nonstem cancer cells,^67^ which helps them resist oxidative stress and evade therapeutic (chemo- and radio-) interventions. Therefore, targeting the redox balance within CSCs by exogenous chemicals capable of elevating the level of ROS is considered a promising strategy for cancer treatment, as it could disrupt their survival advantage.^68,69^ These facts prompted us to verify the efficacy of **RuL2** also on CSCs.

For this experiment, we prepared HCT116 cells in which CSCs were identified and sorted out depending on their expression of the CD133 surface marker.^70^ The effect of **RuL2** on CSC-enriched HCT116.CD133+ subpopulation was then tested and compared to the impact on CSC-depleted HCT116.CD133– cell subpopulation. As indicated (Table 3), the growth of cells was inhibited in both CSC-enriched and CSC-depleted populations in the dark. The effect was markedly enhanced after the cells were irradiated with blue light, the CSC-enriched CD133+ population being slightly but nonsignificantly more sensitive.

**Table 3:** IC_50_ Values (nM)^a^ Obtained for HCT116 CD133+ and CD133– Cells Treated with **RuL2** in the Dark or after Irradiation with Blue (420 nm) Light

	420 nm	dark
HCT116.CD133+	3.3 ± 0.6	286 ± 31
HCT116.CD133–	5 ± 1	260 ± 29

a Data represent a mean ± SD, *n* = 5.

The observation that **RuL2** exhibited roughly equal photoactivity in CSC-depleted and CSC-enriched HCT116 colon cancer cells (Table 3) indicates that it may be equally effective in simultaneously killing both the differentiated and stem cancer cells. This may represent a considerable benefit for the chemotherapy since this attribute may minimize the use of chemotherapeutics specialized to individual types of tumor cells in combination and, importantly, can be targetedly activated by visible light irradiation.

### Effect on 3D Cell Cultures

Three-dimensional (3D) cell cultures exhibit several characteristics of “in vivo” tumors, including hypoxia, cell–cell interactions, and extracellular matrix production/deposition.^71,72^ Moreover, drug penetration also plays an important role. Due to these factors, 3-D cultures of tumor cells are regarded as a more accurate model for in vitro anticancer drug screening.^73,74^ Therefore, the effect of **RuL2** on 3D cultures of HCT116 cells was also tested.

**Figure 7:**
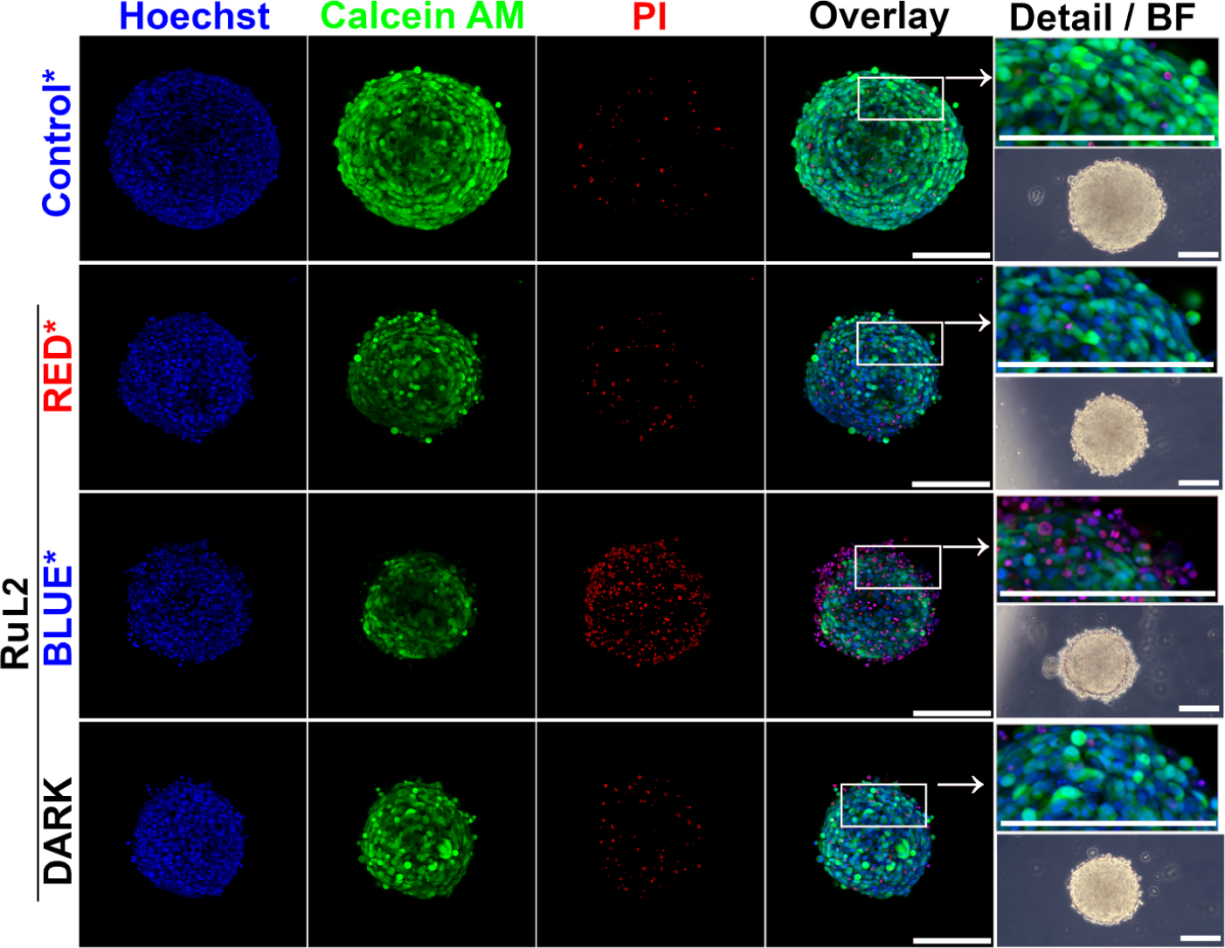
Analysis of HCT116 spheroids by confocal microscopy. Spheroids were treated for 5 h with 1 μM of **RuL2** and subsequently irradiated with blue laser light (1 mW, 180 s). After 67 h post-treatment and irradiation in the drug-free medium, samples were stained with Hoechst 33258 dye, Calcein AM, and propidium iodide. The overlay of fluorescence channels was used to capture spheroid details. Bright-field images were obtained via phase contrast microscopy. Controls were irradiated with blue laser light (405 nm, 180 s), whereas **RuL2**-treated samples were irradiated with blue or red (405 or 650 nm, 180 s) laser light. Both laser lines used for irradiation were adjusted to a power of 1 mW. Scale bars in all panels represent 200 μm. The images represent maximal projections of 3D z-stacks and are representative of two independent experiments performed in triplicate; a quantitative evaluation of all experiments is given in Figure S19.

Spheroids of HCT116 cells (72 h old, average diameter 240 ± 20 nm) were treated with **RuL2** for 5 h in the dark to allow the complex to penetrate the mass of the spheroid. Then, the spheroids were washed, transferred to confocal Petri dishes, and irradiated with 405 nm (blue) or 650 nm (red) laser light for 3 min using lasers included in a confocal microscope. After irradiation, spheroids were cultured in the dark for a further 67 h and subsequently stained with Hoechst 33258, Calcein AM, and PI. Representative samples imaged on a confocal microscope in 10 z-stack scans are shown in Figure 7.

Compared to control spheroids irradiated in the absence of Ru complex, a reduction of the diameter/volume of spheroids is clearly apparent in **RuL2**-treated spheroids (Figure 7). Simultaneously, a decrease in metabolic activity (as manifested by a decrease in Calcein AM fluorescence) and an increase of damaged, PI-positive cells can be observed for spheroids treated with **RuL2**, particularly when the cells were irradiated with blue light. Moreover, peripheral loss of compactness and condensation of the nucleus with nuclear changes, possibly indicating the initiation of apoptosis, are detectable, especially from bright-field images and images showing detailed views, respectively (Figure 7, panels in the rightmost column).

The data show that **RuL2**, if irradiated, can effectively induce cell death even in 3D spheroids, although the enhancement of the effect compared to the nonirradiated sample is less pronounced than in cells cultured in a 2D monolayer. This difference may be due to characteristics unique to 3D cultures and not present in 2D arrangements, such as impaired penetration of Ru complex and/or light to the cells deeper into the inner part of the spheroids. To perceive the effect of light penetration into the spheroid mass, we also used red light, which, thanks to its longer wavelength, can penetrate the tissues deeper.^75^ However, the effect after exposure to red light was lower than that of blue light (Figures 7 and S19), although the power of both lasers was the same (1 mW). Nevertheless, the activity enhancement in 3D culture by blue irradiation was distinct and statistically significant (Figure S19), thus supporting this Ru complex as a potential PDT candidate for further investigation.

## Conclusions

In summary, we designed and synthesized the first substituted benzo[g]quinoxaline-based cyclometalated Ru(II) complexes, [Ru(C^∧^N)(phen)_2_]^+^**RuL1**–**RuL3**, containing a π-expansive cyclometalating C^∧^N ligand that was functionalized at either the aryl or quinoxaline units with an electron-donating group (OMe or NMe_2_) to explore the influence on the absorption properties of the complexes and their photobiological activities. The single-crystal X-ray determination of **RuL1** confirmed the proposed heteroleptic octahedral structure for the new metal complexes. **RuL1**–**RuL3** showed an absorption in the red region of the spectrum and were able to generate singlet oxygen (^1^O_2_) upon red light irradiation in acetonitrile. **RuL1** and **RuL2** could also photogenerate hydroxyl radicals (OH·), a specific type I ROS that could overcome the diminished therapeutic effect in tumor hypoxic microenvironments. No photobleaching was detected during extended irradiation.

Compounds **RuL1**–**RuL3** show dark antiproliferative activity in micromolar concentration in tested cervical, melanoma, and colon human cancer cells. Importantly, they exhibit high phototoxicity after irradiation with light (particularly blue), with the PTI reaching values of 100 for the complex **RuL2** in most sensitive HCT116 cells. The activity of Ru-complexes can also be significantly potentiated by red light, although with lower effectivity. Interestingly, nonirradiated Ru compounds show some selectivity to cancer over noncancerous human cells, suggesting their potential as possible drug candidates for PDT.

The therapeutic usefulness of anticancer agents relies on their ability to exert maximal effect on cancer cells and minimal toxicity to normal cells. When incubated in the dark, the Ru complexes studied here showed selectivity for cancer over noncancerous cells. Nevertheless, their ability to photopotentiate can further augment the difference between the effects on tumor (irradiated) and healthy (nonirradiated) tissue since PDT is applied site-selectively.

The data presented in this paper revealed that **RuL2**, selected as a representative for more detailed biological studies, accumulates in the plasma membrane and membranes of intracellular organelles (mitochondria). If irradiated, it induces lipid peroxidation, likely connected with photoinduced ROS generation. Oxidative damage to the fatty acid chains then leads to the attenuation of the membranes, the activation of caspase 3, and the triggering of the apoptotic pathway, thus realizing membrane-localized PDT.

Currently, the membrane of tumor cells has been recognized as a promising therapeutic target.^76^ The development of novel therapies based on targeting membrane lipids in cancer cells is now being extensively commenced as a new, up-to-date topic.^77^ This approach focuses on destroying cancer cells by damaging their cell membranes instead of binding to specific receptors.^78^ From this point of view, the Ru-complexes reported in this work represent suitable candidate agents due to their selective accumulation and photo-controlled damage to cell membranes.

In summary, the photochemical properties and biological action predispose **RuL2** to become a promising candidate for further studies as a membrane-targeted PDT agent, capable of killing not only the bulk of cancer cells but also the hardly treatable CSCs responsible for tumor recurrence and the metastatic progression of cancer, filling the gap in the use of ruthenium complexes as phototoxic cancer stem cell agents.

## Experimental Section

### Reagents and Chemicals

Synthesis-grade solvents were employed in all cases. Deuterated solvents were purchased from Euriso-top. [Ru(η^6^-*p*-cymene)Cl_2_]_2_, 2,3-diamminenaphtalene, benzyl, 4,4′-dimethoxybenzyl, 4,4′-bis(dimethylammine)benzyl, trifluoroacetic acid, phen, potassium acetate and potassium hexafluorophosphate were obtained from Merck (Madrid, Spain. The purities ≥95% of the synthesized complexes used for biological evaluation were determined by RP-HPLC.

A description of the synthesis of the compounds herein investigated can be found in the Supporting Information.

### X-ray Structure Determinations

Intensities were registered at low temperatures on a Bruker D8QUEST diffractometer using monochromated Mo *K*α radiation (λ = 0.71073 Å). Absorption corrections were based on multiscans (program SADABS).^79^ Structures were refined anisotropically using SHELXL-2018.^80^ Hydrogen atoms were included using a riding model.

*Unique Features of***RuL1**. The structure contains one dichloromethane molecule disordered over two positions, ca. 69:315.

### Microwave

The ruthenium complexes were synthesized in an Anton Paar Monowave 50 (315 W) microwave.

### Nuclear Magnetic Resonance (NMR) Spectroscopy

The ^1^H, ^13^C{^1^H}, and bidimensional NMR spectra were recorded on a Bruker AC 300E, Bruker AV 400, or Bruker AV 600 NMR spectrometer, and chemical shifts were determined by reference to the residual ^1^H and ^13^C{^1^H} solvent peaks.

### Elemental Analysis

The C, H, N, and S analyses were performed with a Carlo Erba model EA 1108 microanalyzer with EAGER 200 software.

### Mass Spectrometry (MS)

ESI mass (positive mode) analyses were performed on an RP/MS TOF 6220. The isotopic distribution of the heaviest set of peaks matched very closely to that calculated for formulating the complex cation in every case.

### Photophysical Characterization

UV/vis spectroscopy was performed on a PerkinElmer Lambda 750 S spectrometer with operating software. Solutions of all complexes were prepared in acetonitrile and water (1% DMSO) at 10 μM.

### Stability

To check the stability of the compounds, their UV/vis spectra were recorded in DMSO (10 μM) at different at *t* = 0 and after the incubation for 48 h at 37 °C.

*Photostability.* The photostability of the compounds was checked by recording their UV/vis spectra in DMSO (10 μM) before and after 2 h of blue (465 nm, 5 mW cm^–2^) and red light irradiation (620 nm, 15 mW cm^–2^).

### Singlet Oxygen Quantum Yield

Singlet oxygen quantum yields were calculated in aerated acetonitrile solution using DPBF as a chemical trap upon red light irradiation (620 nm, 5.06 mW cm^–2^) using methylene blue as a reference. Photolysis of DPBF in the presence of ruthenium complexes was monitored by UV/vis, the absorbance of DPBF at 411 was plotted against irradiation times, and slopes were calculated. Finally, singlet oxygen quantum yields were calculated using the following equation:
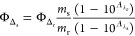
where Φ_Δ_r__ is the singlet oxygen quantum yield of the reference, as said methylene blue (Φ_Δ_r__ = 0.60 in acetonitrile); *m*_s_ and *m*_r_ are the slopes of the complex and the reference, respectively; and *A*_λ_s__ and *A*_λ_r__ are the absorbances of the compound and reference at the irradiation wavelength (620 nm), respectively.

### Hydroxyl Radical Generation in Cell-Free Media

All compounds (10 μM) were prepared in PBS (5% DMF). To this solution, HPF was added with a final concentration of 10 μM. Then, samples were irradiated by red light (620 nm, 20 mW cm^–2^) for indicated time intervals. Fluorescence spectra were obtained with a Horiba Jobin Yvon Fluorolog 3–22 modular spectrofluorometer with a 450 W xenon lamp. Measurements were performed in a right-angled configuration using 10 mm quartz fluorescence cells for solutions at 298 K. The excitation wavelength was set to 490 nm, and the excitation and emission slit widths were 3 nm.

### Cell Lines and Culture Conditions

HeLa human cervix adenocarcinoma cells and A375 human skin melanoma cells were purchased from ECACC (UK). HCT116 and MRC5pd30 were obtained from ATCC, Manassas, VA, USA. Cells were cultured in DMEM growth medium (high glucose, 4.5 g L^–1^, Biosera) supplemented with gentamycin (50 mg mL^–1^) and 10% inactivated FBS (Biosera); media for the MRC5 cells were fortified by 1% nonessential amino acids (Sigma-Aldrich, Prague, Czech Republic).

For the biological experiments, the stock solutions of Ru complexes were prepared in DMSO and further diluted to the EBSS or DMEM medium as needed. The final concentration of DMSO in biological experiments did not exceed 0.1%. It was verified that this DMSO concentration did not affect cells’ viability.

### Treatment and Irradiation of Cells

The cells were seeded on cell culture plastic (96-well plates or Petri dishes) in DMEM and cultured overnight in a humidified atmosphere (37 °C, 5% CO_2_). Then, the medium was removed, cells were washed, and the tested compound diluted in EBSS was added. The cells were incubated for 1 h in the dark (37 °C, 5% CO_2_) and subsequently irradiated for 1 h at 37 °C with blue or red light (or kept in the dark). The cells were irradiated using an LZC-4 photoreactor (Luzchem Research, Gloucester, Canada) equipped with 16 lamps LZC-420 with a maximum centered at 420 nm (blue light) or with 16 LZC-cool white lamps covered with a red filter to select a specific wavelength range λ_max_ = 613 nm. An average blue and red light irradiance was 58 and 20 W m^–2,^ respectively, as measured using a Light Meter LI-250A with a quantum sensor (LI-COR, Nebraska, USA)]. Control cells were incubated and irradiated with Ru-free EBSS containing the same concentration of DMSO (<0.1%) as in the cells treated with Ru complexes. After the irradiation, EBSS containing Ru compounds was removed, and cells were further incubated in a complete drug-free DMEM culture medium for the indicated time.

### Phototoxicity Testing

The cells seeded in a 96-well plate were treated with Ru complexes diluted in EBSS (1 h) and irradiated (1 h) as described above. After the irradiation, the Ru-containing EBSS was removed, and cells were allowed to recover for a further 70 h. The number of live cells was determined using a standard MTT or SRB assay. The IC_50_ values were obtained from dose–response curves. The PTI was calculated as a ratio of IC_50_ (dark)/IC_50_ (irradiated).

### Intracellular Ru Accumulation and Subcellular Localization

The amount of Ru taken up by HCT116 cells treated with tested compounds at their equimolar concentrations (3 μM in EBSS) for 2 h at 37 °C in the dark was measured as already described^81,82^ by ICP-MS (Agilent Technologies, CA, USA). The concentrations of Ru in the samples were related to the amount of cells in the sample, determined using TC10 Automated Cell Counter) (Biorad). To assess the distribution of Ru in cells, the cells were fractionated into four fractions (cytosolic, membrane/particulate, nuclear, and cytoskeletal) using the FractionPREP Cell Fractionation kit (BioVision) according to the manufacturer’s instructions. Each fraction was freeze-dried, resuspended in 200 μL of 35% HCl, and mineralized. Samples were diluted in water, and Ru concentration was determined by ICP-MS.

### ROS Detection in Cells

HCT116 cells were treated with indicated concentrations of Ru complex and irradiated as indicated (*vide supra*). Immediately after irradiation, the cells were washed and stained with 5 μM CellROX Green (Invitrogen) in PBS for 30 min at 37 °C. Next, the cells were washed with PBS and harvested. Fluorescence was measured using flow cytometry.

### Morphology Studies

HCT116 cells were seeded at the density of 1 × 10^5^ cells per confocal dish (35 mm; Mattek). After the overnight incubation, the culture medium was replaced by EBSS, and cells were treated with **RuL2** (80 nM) or the respective vehicle (DMSO) control. Samples were kept in the dark for 1h in a humidified CO_2_ incubator and consecutively irradiated with blue light (420 nm). Then, the Ru-containing medium was removed, and cells were further incubated in Ru-free DMEM medium at 37 °C. Samples were observed and imaged under an inverted microscope Olympus CKX41. Alternatively, samples were washed with PBS and stained with Hoechst 33342 (8 μM) for 15 min. Samples were washed, and the staining solution was replaced with the DMEM culture medium without phenol red. The samples were then analyzed using a Leica CM SP5 confocal microscope. Regions of interest were exposed with 405 nm blue laser light (60 s, 1 mW). Samples were analyzed for up to 150 min postirradiation.

### Caspase 3 Activity Assay

The activation of caspase 3 was detected using CellEvent Caspase 3/7 Green - Active Caspase 3/7 Assay Kit (Thermo Fisher Scientific). Briefly, HCT116 cells were seeded at a 6-well plate at 2.5 × 10^5^ cells/well density and treated and irradiated as described above (1 h preincubation in the dark, 1 h irradiation at 420 nm). After 2 h of recovery in compound-free media, cells were stained with the CellEventCaspase 3/7 Green Detection Reagent according to the manufacturer’s protocol, and the fluorescence signal was analyzed by flow cytometry.

### Cell Membrane Labeling and Imaging

HCT116 cells were seeded at the density of 1 × 10^5^ cells per confocal dish (35 mm; Mattek). After the overnight incubation, the culture medium was replaced by EBSS, and cells were treated with **RuL2** (80 nM) or the respective vehicle (DMSO) control. Samples were kept in the dark for 120 min in a humidified CO_2_ incubator. Then, samples were washed with PBS and stained with TMRE (100 nM), Hoechst 33342 (8 μM), and CellMask deep red (according to the manufacturer protocol) for 15 min. Samples were washed, and the staining solution was replaced with the DMEM culture medium without phenol red. The samples were then analyzed sequentially using a Leica CM SP5 confocal microscope. Regions of interest were exposed with 405 nm blue laser light (60 s, 1 mW).

### Lipid Peroxidation

HCT116 cells seeded on the 6-well plate at the density of 2.5 × 10^5^ cells/well were treated with an indicated concentration of **RuL2** in the dark for 1 h and then irradiated with blue light (420 nm) for 1 h. As a positive control, menadiol (100 μM) was employed (2 h of incubation in the dark). After the treatment, the medium containing tested compounds was removed, and samples were stained with Bodipy 665/676 dye (ThermoFisher Scientific) at the final concentration of 5 μM in FBS/phenol red-free medium and incubated for 30 min in a humidified CO_2_ incubator. Cells were then washed with PBS, harvested, and analyzed by flow cytometry (BD FACS Verse). Data were analyzed using FCS Express 6 (DeNovo software; Glendale, CA).

### Phototoxicity in CSCs

CSC-enriched (HCT116.CD133+) and CSC-depleted (HCT116.CD133−) cell populations were prepared by cell sorting. The HCT116 cells were stained for their surface CSC marker CD133 with CD133/1-APC (Miltenyi Biotec, Reutlingen, Germany) for 10 min at 4 °C. Subsequently, the cells were washed and labeled with anti-APC microbeads for 15 min at 4 °C. After washing, the cells were magnetically sorted on an LS column placed between magnets on the MACS stand. Two fractions, CD133 positive (CD133+) and CD133 negative (CD133−) were obtained. The quality of the cell distribution to the two populations was further verified by FACS. The sorted cells were stained with CD133/1-APC antibody for 10 min at 4 °C. After washing, the cells were analyzed on flow cytometer BD FACS Verse to confirm positivity/negativity for the CD133 marker. Sorted HCT116.CD133+ and HCT116.CD133– cells were seeded at 96-well plates, incubated with **RuL2**, and irradiated (or kept in the dark) as described above. The phototoxic effect of **RuL2** was determined by MTT assay 70 h after irradiation.

### Effect on 3D-Cell Culture

HCT116 cells were seeded on 96w ultralow attachment U-shape plates (Corning) at the density of 500 cells/well in the 3D forming medium: DMEM-F12 ham medium supplemented with growth and spheroid forming factors: 2% B27 (Thermo Fisher Scientific Inc., MA, USA), epidermal growth factor (EGF; Sigma-Aldrich, Germany, 20 ng mL^–1^), fibroblast growth factor (FGF2; Sigma-Aldrich, Germany, 10 ng mL^–1^) and bovine serum albumin (BSA) (Sigma-Aldrich, Germany, 0.15%). After 72 h of incubation, preformed spheroids were transferred as single spheres and treated with tested compounds at the concentration of 1 μM for 5 h, and following that, the spheroids were washed and transferred to confocal 35 mm Petri dishes (Mattek). Samples determined for irradiation were irradiated with 405 nm (blue) of 650 nm (red) laser light for 3 min at the final power of 1 mW using confocal microscope Leica CM SP5 (Leica, Germany). Spheroids were cultured for a further 67 h postirradiation and, after this period, were processed for further staining with Hoechst 33258 (20 μg mL^–1^), Calcein AM (2 μM), and PI 8 μg mL^–1^ for 2 h. Samples were imaged on a confocal microscope Leica CM SP8 SMD in 10 z-stack scans. Images were processed by ImageJ software.

## Supporting Information Available

Molecular formula strings and biological data (CSV)
Synthesis of the compounds; NMR spectra; high-performance liquid chromatography and mass spectrometry; X-ray crystallographic analysis for **RuL1**; photophysical properties; stability and photostability; evaluation of ^1^O_2_ photogeneration; biological experiments: IC_50_ values determined by the SRB assay; the efficiency to generate ROS; quantification of Calcein AM analyzed from the confocal microscopy microphotographs (PDF)

## Abbreviations

3D: three-dimensional
BSA: bovine serum albumin
CSC: cancer stem cell
DMEM: Dulbecco’s modified Eagle’s medium
DMSO: dimethyl sulfoxide
dpbf: 1,3-diphenylisobenzofuran
dpbq: diphenylbenzo[g]quinoxaline
EBSS: Earle′s balanced salts solution
FACS: fluorescence-activated cell sorting
FBS: fetal bovine serum
HPF: 3′-*p*-(hydroxyphenyl)fluorescein
IC_50_: concentration of the agent inhibiting cell growth by 50%
ICD: immunodenic cell death
ICP-MS: inductively coupled plasma mass spectrometry
MTT: 3-[4,5-dimethylthiazol-2-yl]-2,5 diphenyl tetrazolium bromide
NMR: nucler magnetic resonance
PBS: phosphate buffered saline
PDT: photodynamic therapy
PI: propidium iodide
PTI: phototoxicity index
RPMI: Roswell Park Memorial Institute
ROS: reactive oxygen species
SD: standard deviation
SI: selectivity index
SRB: Sulforhodamine B
TMRE: tetramethylrhodamine ethyl ester
