# Supplementary material for: A Novel Substituted Benzo[g]quinoxaline-Based Cyclometalated Ru(II) Complex as a Biocompatible Membrane-Targeted PDT Colon Cancer Stem Cell Agent
Source: J Med Chem. 2024 Dec 2;67(23):21470–85. doi: 10.1021/acs.jmedchem.4c02357 (PMC11647881; doi:10.1021/acs.jmedchem.4c02357)
Supplement: Supplementary file 2 — jm4c02357_si_002.pdf [file jm4c02357_si_002.pdf]

SUPPORTING INFORMATION

A novel substituted benzo[*g*]quinoxaline-based cyclometalated Ru(II) complex as a biocompatible membrane-targeted PDT colon cancer stem cell agent

Alicia Marco,[‖a] Jana Kasparkova,[‖b,c] Delia Bautista,[d] Hana Kostrhunova,[b] Natalia Cutillas,[a] Lenka Markova,[b] Vojtech Novohradsky,[b] José Ruiz*[a] and Viktor Brabec*[,b,c]

[a]*Departamento de Química Inorgánica, Universidad de Murcia, and Murcia BioHealth Research Institute (IMIB-Arrixaca), E-30100 Murcia, Spain*
[b]*Czech Academy of Sciences, Institute of Biophysics, Kralovopolska 135, CZ-61200 Brno, Czech Republic*
[c]*Department of Biophysics, Faculty of Science, Palacky University, Slechtitelu 27, 783 71 Olomouc, Czech Republic*
[d]*ACTI, Universidad de Murcia, Murcia E-30100, Spain*

*Corresponding authors' e-mail addresses:
e-mails: jruiz@um.es (J. Ruiz), vbrabec44@gmail.com (V. Brabec)

1. Synthesis of the compounds S3
2. NMR spectra S5
3. High perfomance liquid chromatography and mass spectrometry....................S12
4. X-Ray crystallographic analysis for **RuL1** S15
5. Photophysical properties....................S18
6. Stability and photostability....................S19
7. Evaluation of $^1O_2$ photogeneration S21
8. Biological experiments S21
9. Reference....................S23

1. Synthesis of the compounds

*Synthesis of quinoxaline-based proligands (**HL1–HL3**).*

The preparation of proligands **HL1** and **HL2** was carried out as previously reported [1]. The new proligand **HL3** was prepared in a similar manner (see Scheme S1).

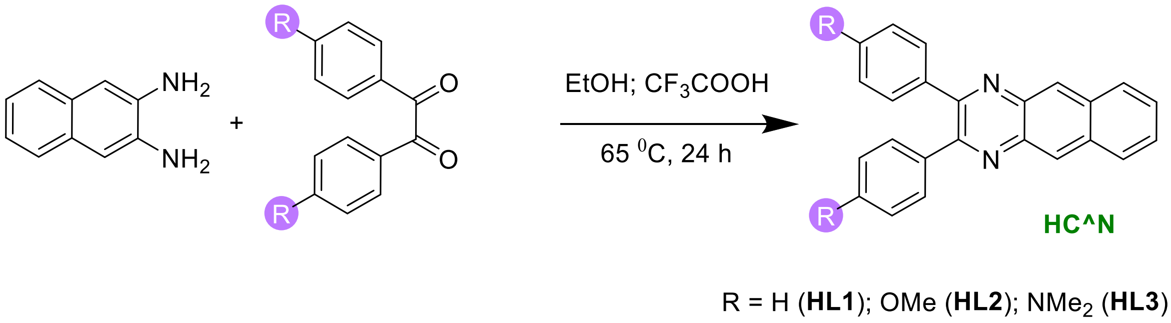

Scheme S1. Synthetic procedure for **HL1–HL3**.

Data of HL3: ^{1}H NMR (600 MHz, $CDCl_3$) δ 8.61 (s, 2H), 8.06 (dd, *J* = 6.4, 3.3 Hz, 2H), 7.57 (d, *J* = 9.0 Hz, 4H), 7.50 (m, 2H), 6.69 (d, *J* = 9.0 Hz, 4H), 3.01 (s, 12H). ^{13}C{^{1}H} NMR (151 MHz, $CDCl_3$): δ 154.6, 151.0, 138.3, 133.7, 131.1, 128.6, 127.6, 126.8, 126.1, 111.8, 40.4. ESI-MS (positive ion mode): *m*/*z* = 419.2230 $[M + H]^+$ calcd *m*/*z* 419.2235. Anal. Calcd for $C_{28}H_{26}N_4$: %C, 80,16; %H, 6.49; %N, 13.35. Experimental: %C, 80.21; %H, 6.43; %N, 13.22.

Preparation of new Ru(II) complexes

$[Ru(h^6\text{-}p\text{-cymene})Cl_2]_2$ (0.1 mmol), potassium acetate (0.6 mmol), KPF_6 (0.8 mmol) and the corresponding HC^N pro-ligand (0.2 mmol), previously synthesized, were added to a microwave tube, dissolved in acetonitrile (5 mL) and stirred at 90 °C for 3 h. The solvent was then removed under reduced pressure and the crude product was dissolved in methanol (5 mL). Then, phenanthroline (0.4 mmol) was added to the solution and stirred at 90 °C for 3 h in a microwave tube. The solvent was removed under reduced pressure, and the resultant violet solid was further purified by column chromatography on alumina with a mixture of DCM:ACN (8:2).

Data of RuL1: yield 35%. ^{1}H-NMR (400 MHz, CD_3CN) δ 8.64 (s, 1H), 8.53 – 8.48 (m, 2H), 8.47 – 8.42 (m, 4H), 8.41 – 8.36 (m, 2H), 8.25 – 8.16 (m, 2H), 8.05 – 7.99 (m, 2H), 7.96 (d, *J* = 8.3 Hz, 1H), 7.85 – 7.43 (m, 4H), 7.66 – 7.59 (m, 4H), 7.49 – 7.44 (m, 1H), 7.44 – 7.39 (m, 2H), 7.38 – 7.31 (m, 1H), 7.27 – 7.23 (m, 1H), 7.22 – 7.18 (m, 1H), 6.58 – 6.48 (m, 2H), 6.28 – 6.20 (m, 1H). ^{13}C{^{1}H} (101 MHz, CD_3CN) δ 203.0, 165.0, 156.0, 154.7, 153.8, 151.3, 150.8, 150.0, 148.8, 148.4, 148.0, 146.3, 142.3, 142.2, 138.1, 137.2, 136.4, 136.0, 135.4, 134.8, 134.1, 134.0, 133.0, 131.7, 131.6, 131.6, 131.1, 130.4, 130.1, 129.8, 128.9, 128.8, 128.7, 128.7, 128.6, 128.5, 128.4, 128.2, 128.0, 127.6, 126.7, 126.4, 126.3, 126.0, 122.5, 120.9. ESI-MS (positive ion mode, ACN): *m*/*z* = 793.1648 $[M\text{-}PF_6]^+$, calcd 793.1674. Anal. Calcd. for $C_{48}H_{31}F_6N_6PRu$: %C, 61.47; %H, 3.33; %N, 8.96. Found: %C, 61.45; %H, 3.51; %N, 8.81.

Data of RuL2: yield 40%. ^{1}H-NMR (600 MHz, CD_3CN) δ 8.53 – 8.49 (m, 3H), 8.47 – 8.44 (m, 2H), 8.44 – 8.41 (m, 2H), 8.40 – 8.36 (m, 2H), 8.23 – 8.16 (m, 2H), 8.05 – 8.00 (m, 2H), 7.93 – 7.89 (m, 1H), 7.79 (dd, *J* = 8.1, 4.8 Hz, 1H), 7.77 – 7.72 (m, 3H), 7.64 (dd, *J* = 5.2, 1.2 Hz, 1H), 7.47 (dd, *J* = 8.0, 5.5 Hz, 1H), 7.42 (dd, *J* = 8.2, 5.3 Hz, 1H), 7.40 – 7.36 (m, 1H), 7.33 – 7.29 (m, 1H), 7.28 (d, *J* = 9.0 Hz, 1H), 7.15 – 7.11 (m, 3H), 6.15 (dd, *J* = 9.0, 2.6 Hz, 1H), 5.67 (d, *J* = 2.7 Hz, 1H), 3.91 (s, 3H), 3.27 (s, 3H). ^{13}C{^{1}H} (151 MHz, CD_3CN) δ 206.0, 164.3, 161.7, 159.4, 156.0, 154.3, 153.8, 151.4, 150.8, 150.0, 148.9, 148.7, 146.3, 142.1, 140.5, 138.0, 137.1, 135.8, 135.3, 134.7, 134.6, 134.5, 134.0, 133.6, 131.8, 131.6, 131.5, 131.4, 131.2, 128.8, 128.7, 128.6, 128.5, 128.5, 128.4, 128.3, 127.7, 127.2, 126.7, 126.4, 126.3, 126.0, 122.0, 120.0, 115.0, 107.9, 56.2, 54.9. ESI-MS (positive ion mode, ACN): *m/z* = 853.1859 $[M\text{-}PF_6]^+$, calcd = 853.1893 . Anal. Calcd. for $C_{50}H_{35}F_6N_6O_2PRu$: %C, 60.18; %H, 3.54; %N, 8.42. Found: %C, 60.14; %H, 3.54; %N, 8.30.

Data of RuL3: yield 37%. ^{1}H-NMR (400 MHz, CD_3CN) δ 8.56 – 8,48 (m, 4H), 8.44 – 8.34 (m, 3H), 8.31 (s, 1H), 8.26 (s, 1H), 8.20 – 8.12 (m, 2H), 8.07 – 8.00 (m, 2H), 7.89 – 7.84 (m, 1H), 7.81 – 7.72 (m, 2H), 7.69 – 7.65 (m, 2H), 7.64 (dd, *J* = 5.4, 1.2 Hz, 1H), 7.46 – 7.42 (m, 1H), 7.41 – 7.36 (m, 1H), 7.35 – 7.29 (m, 1H), 7.29 – 7.22 (m, 2H), 7.08 – 7.04 (m, 1H), 6.85 (d, *J* = 8.9 Hz, 2H), 5.92 (dd, *J* = 9.2, 2.7 Hz, 1H), 5.35 (d, *J* = 2.7 Hz, 1H), 3.04 (s, 6H), 2.41 (s, 6H). ^{13}C-RMN (151 MHz, CD_3CN) δ 204.6, 164.1, 156.0, 155.0, 153.6, 152.4, 151.4, 150.8, 150.1, 149.4, 149.1, 146.4, 142.2, 138.2, 136.8, 135.5, 135.4, 134.7, 134.2, 134.1, 133.9, 133.0, 131.7, 131.5, 131.3, 131.2, 131.1, 129.6, 128.7, 128.5, 128.4, 128.3, 128.0, 127.7, 127.3, 126.6, 126.5, 126.2, 126.1, 125.8, 121.0, 117.2, 112.5, 106.6, 40.4, 39.3. ESI-MS (positive ion mode, ACN): *m/z* = 879.2498 $[M\text{-}PF_6]^+$ calcd ; m/z = 879.2515 . Anal. Calcd. for $C_{52}H_{41}F_6N_8PRu$: %C, 60.99; %H, 4.04; %N, 10.94. Found: %C, 60.98; %H, 4.07; %N, 11.01.

2. NMR spectra

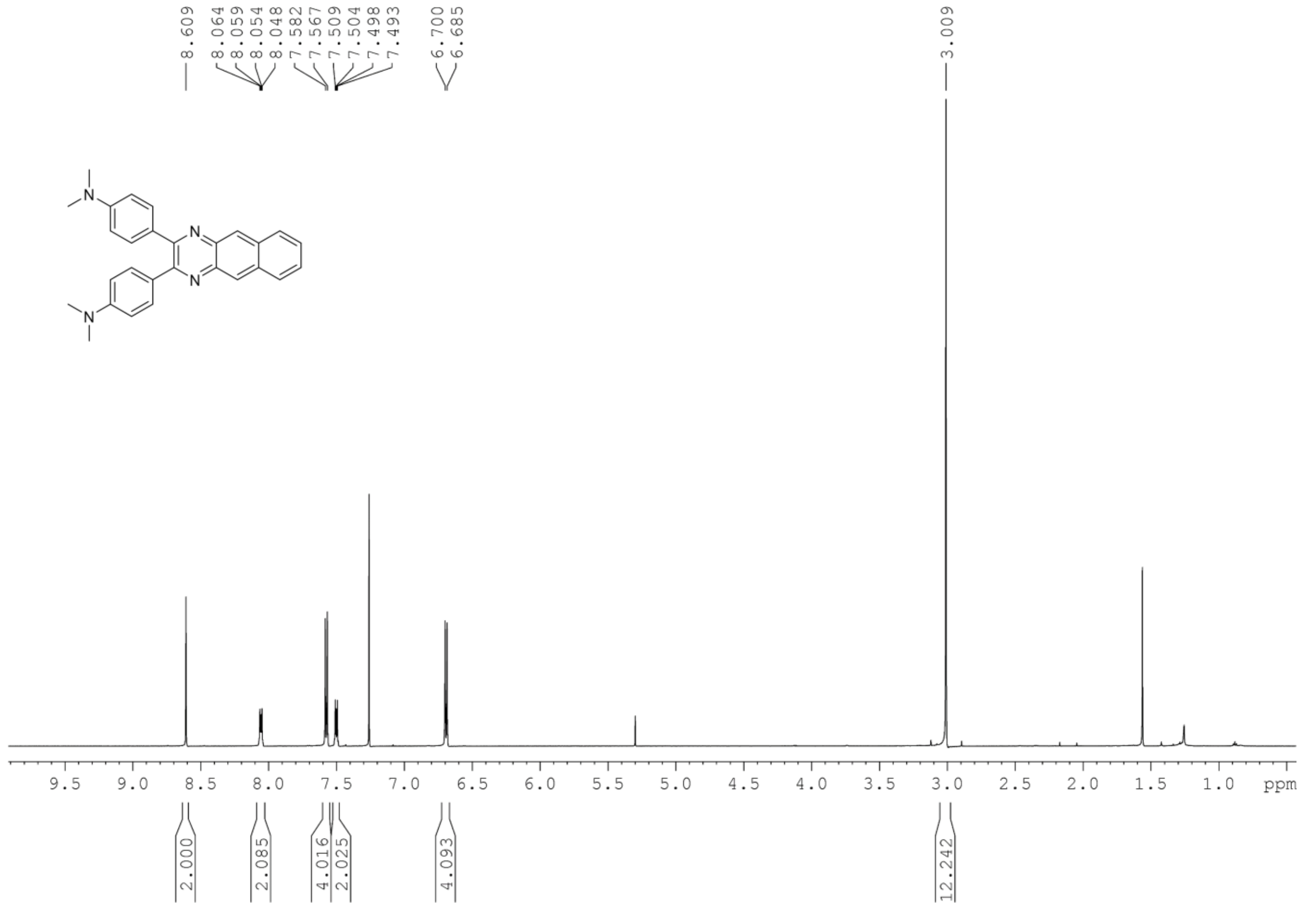

Figure S1. ^{1}H NMR spectra of compound **HL3** in $CDCl_3$.

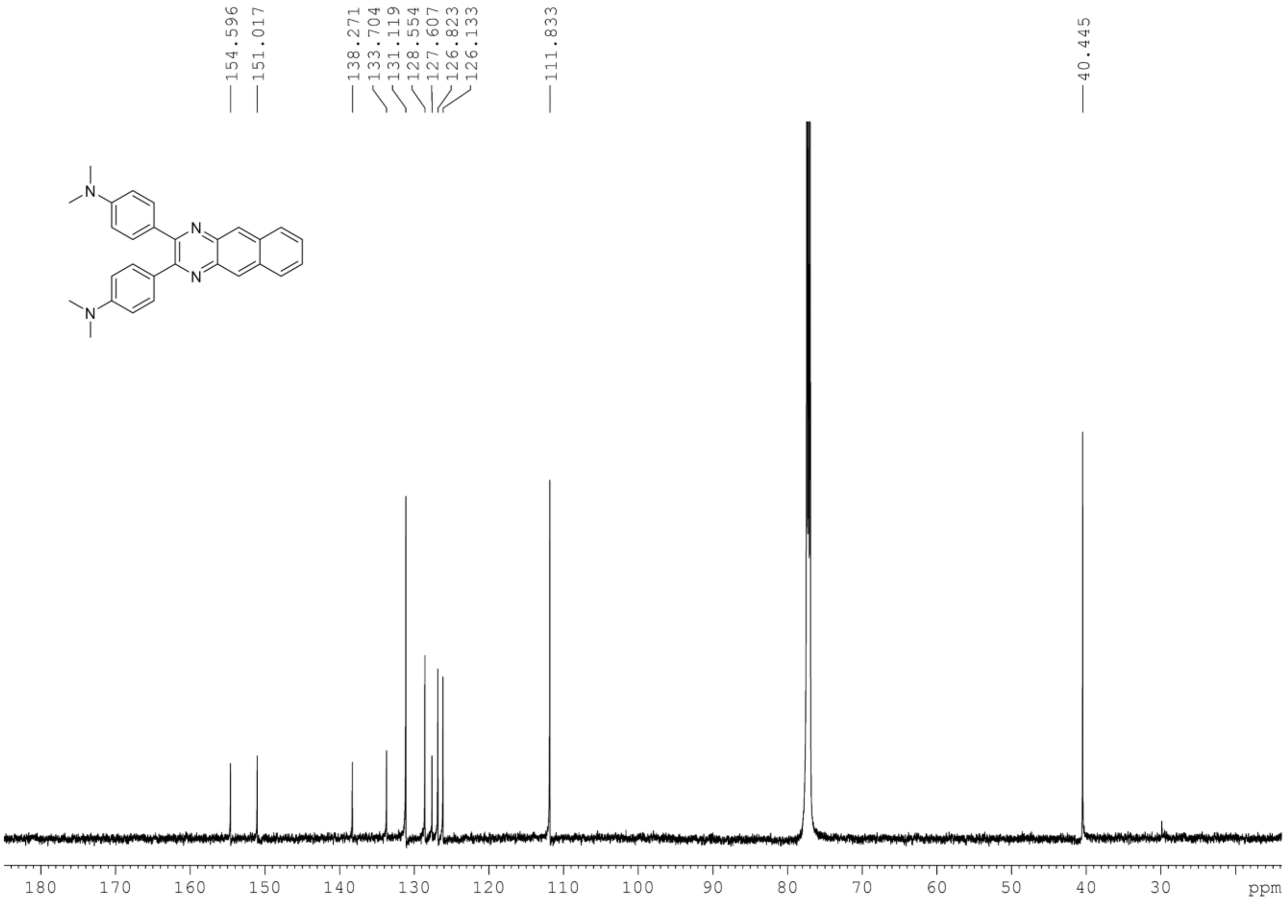

Figure S2. ^{13}C NMR spectra of compound **HL3** in $CDCl_3$.

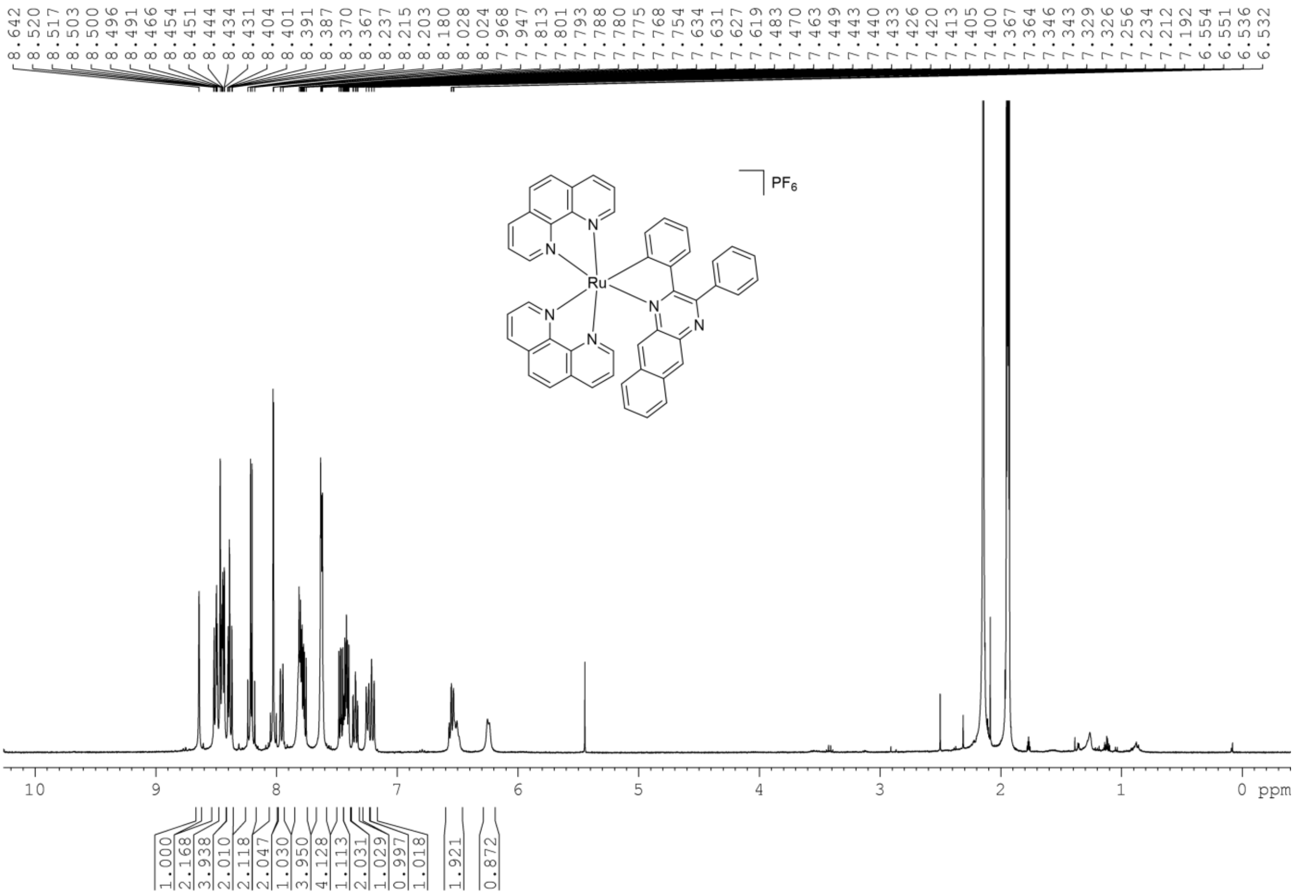

Figure S3. ^{1}H NMR spectra of compound **RuL1** in CD_3CN.

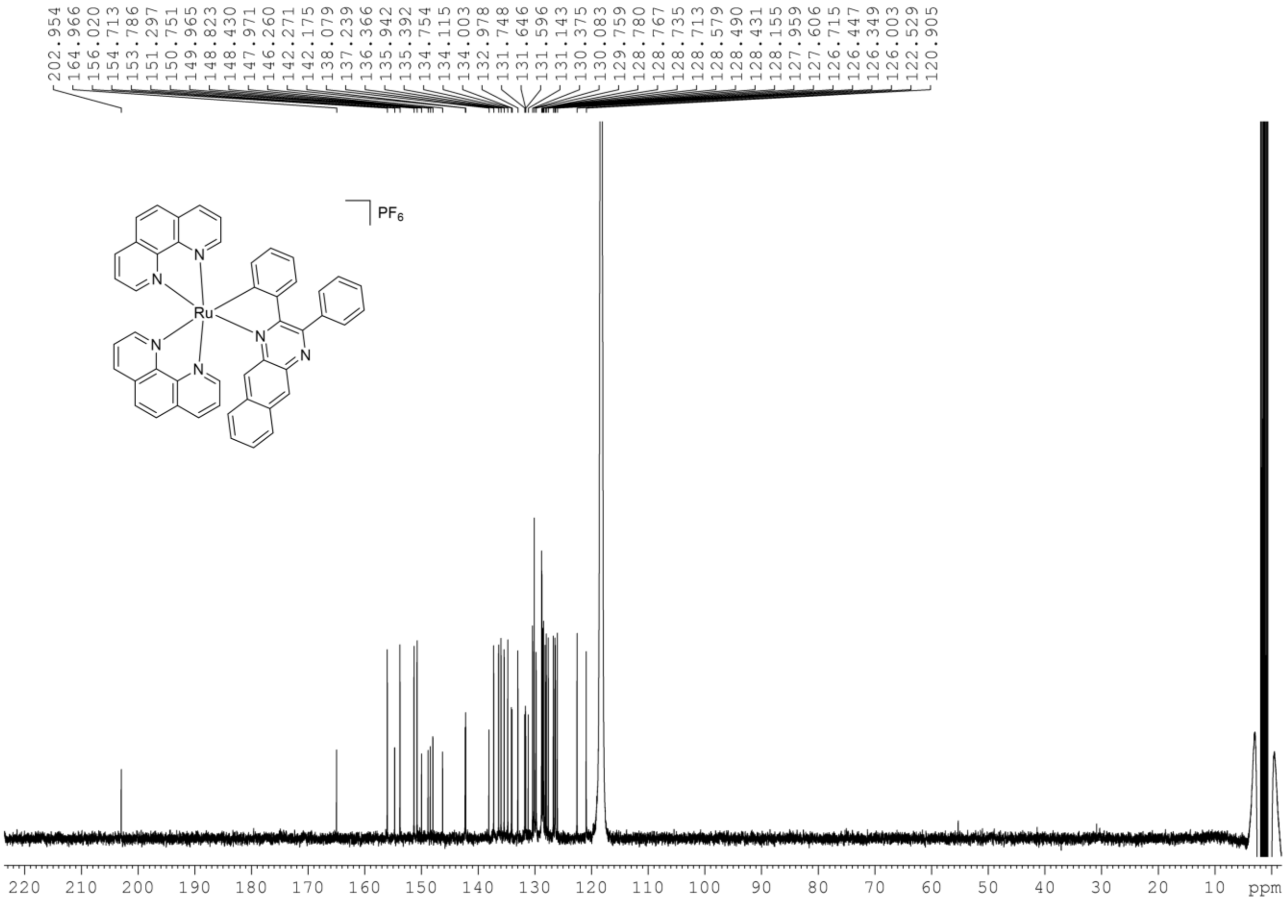

Figure S4. ^{13}C NMR spectra of compound **RuL1** in CD_3CN.

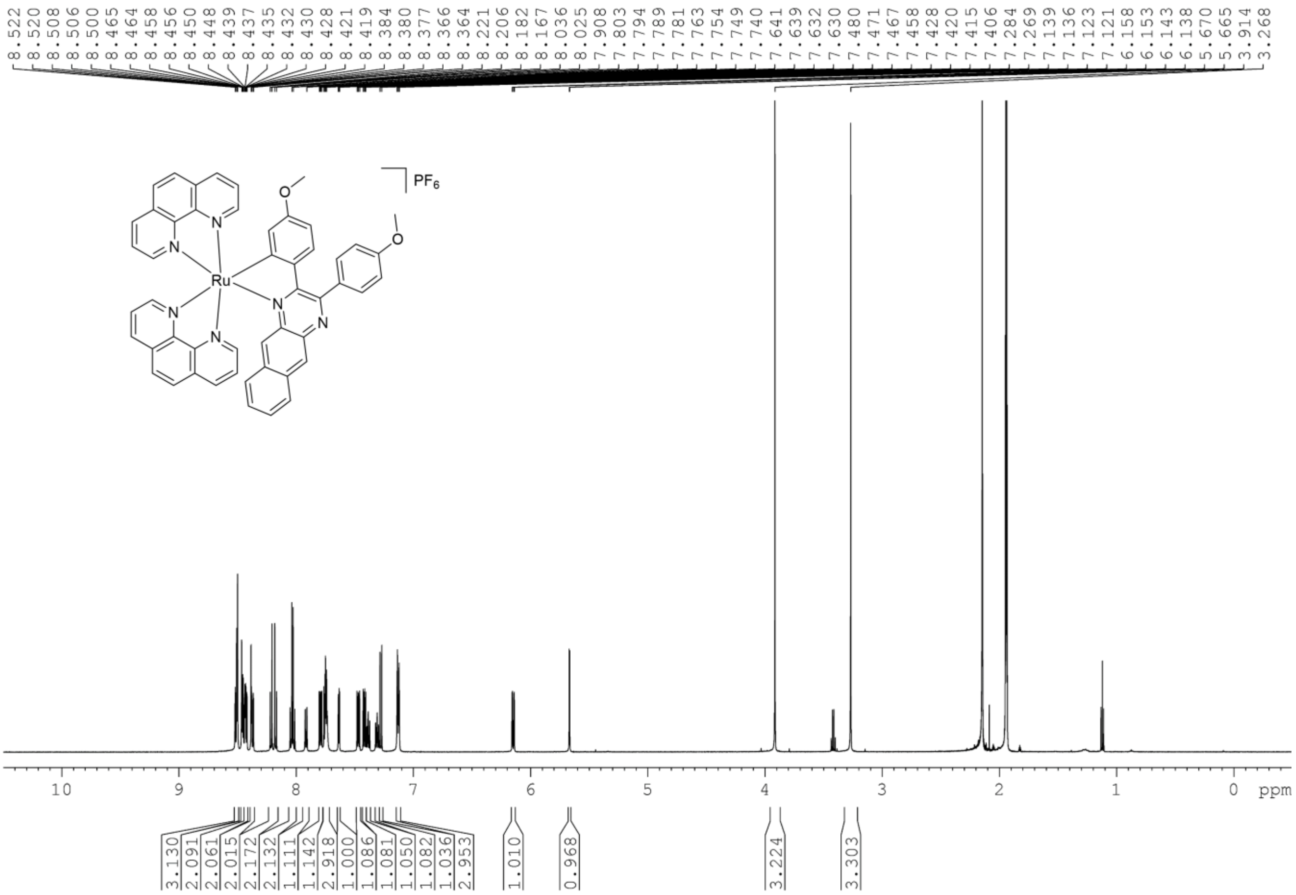

Figure S5. ^1H NMR spectra of compound **RuL2** in CD_3CN.

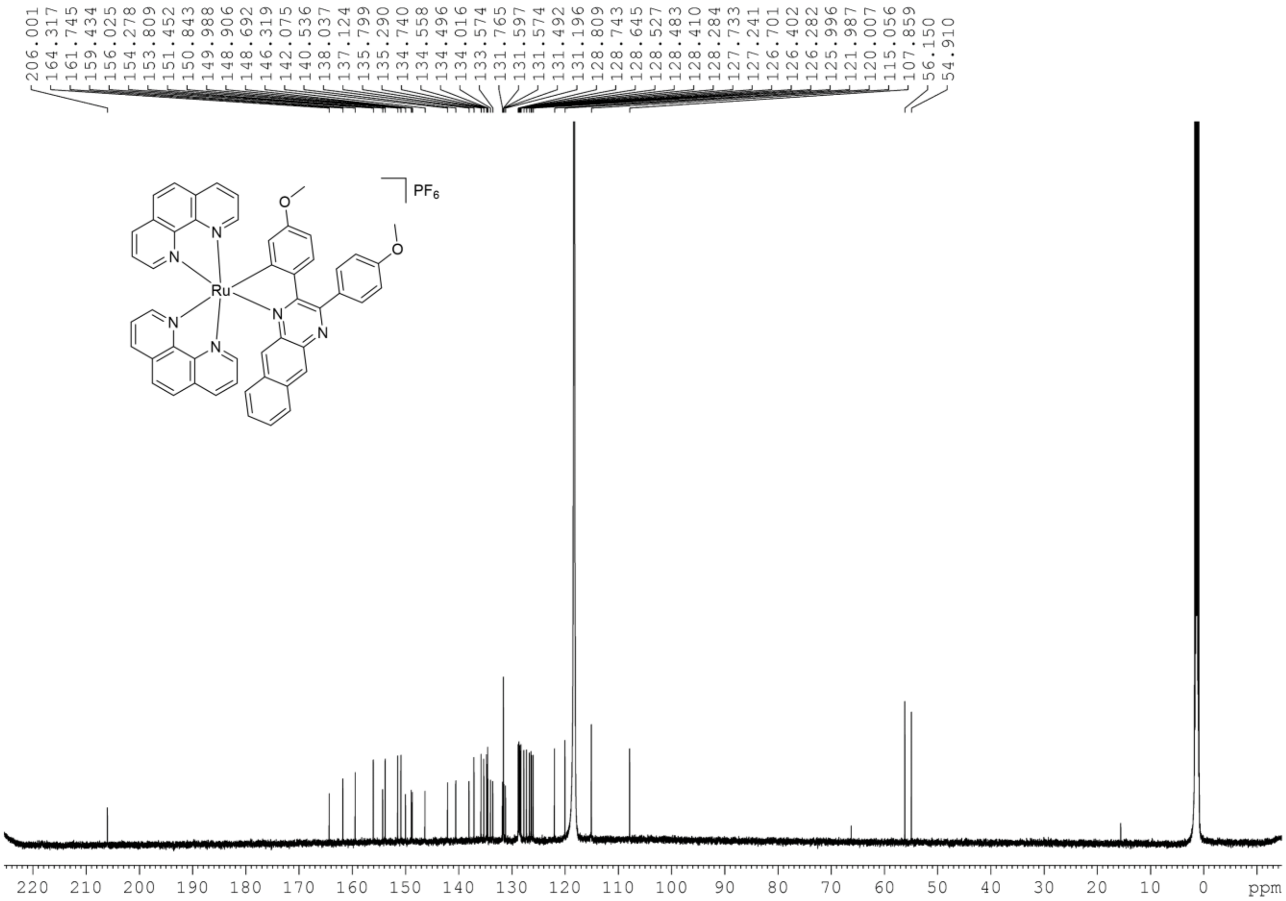

Figure S6. ^{13}C NMR spectra of compound **RuL2** in CD_3CN.

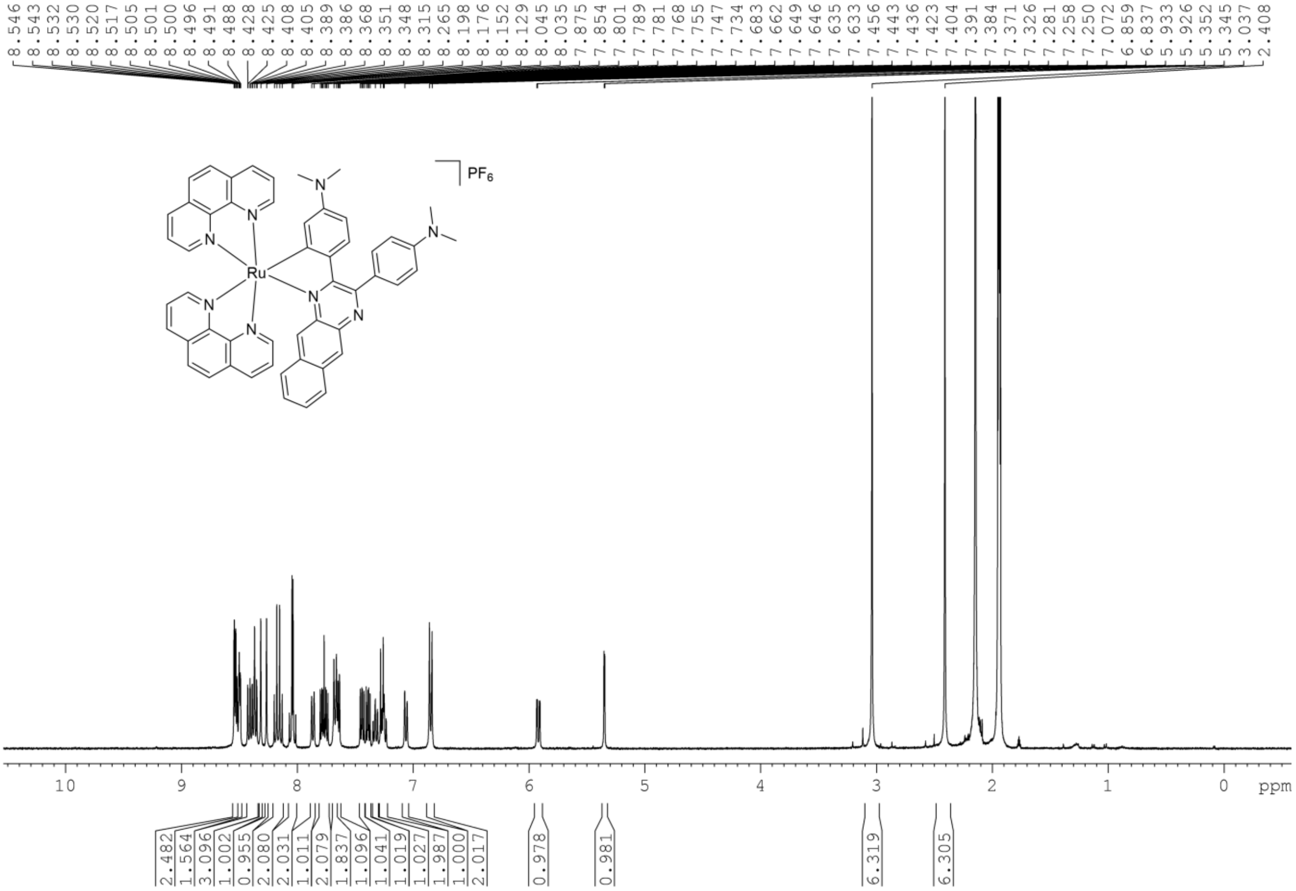

Figure S7. ^{1}H NMR spectra of compound **RuL3** in CD_3CN.

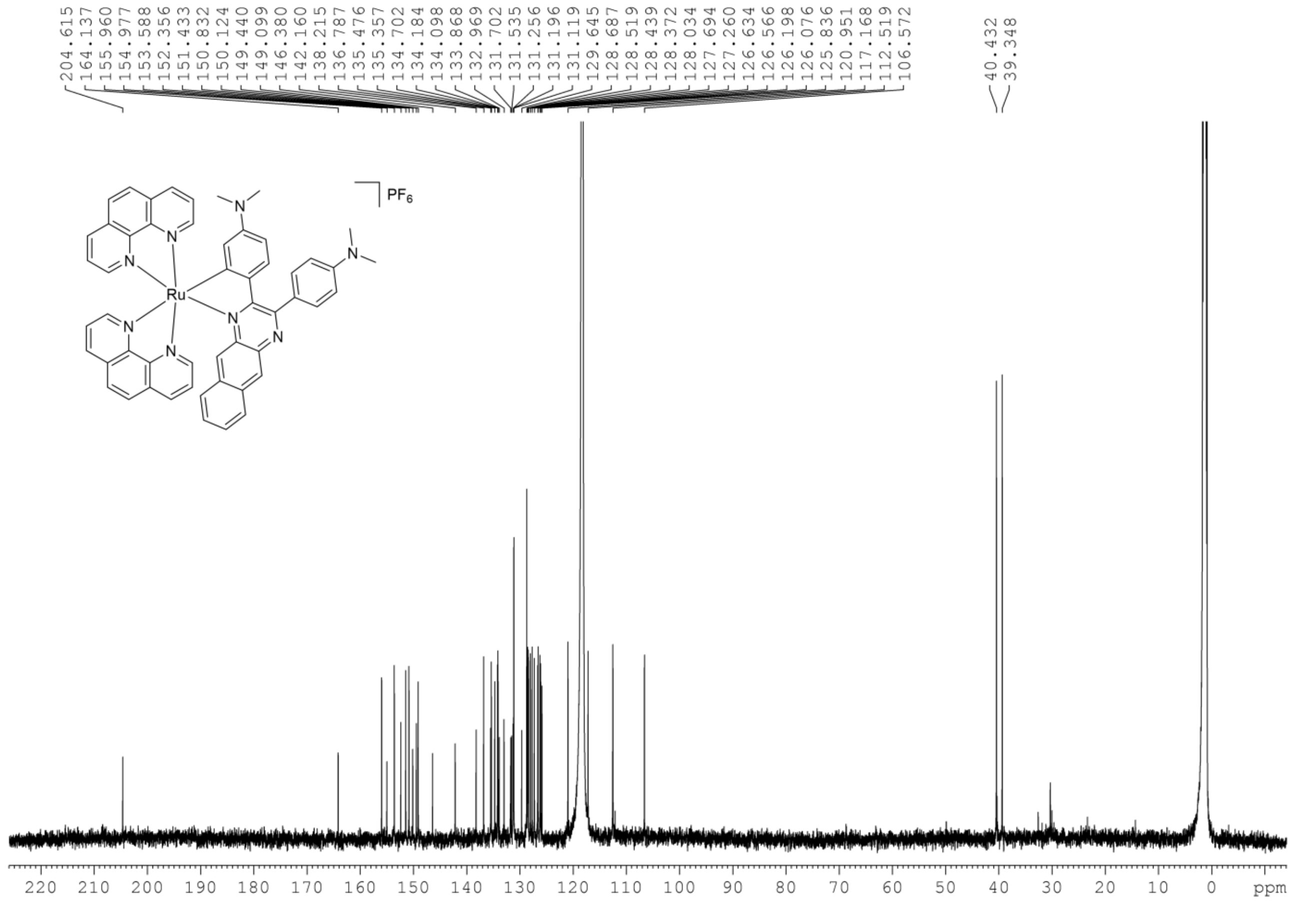

Figure S8. ^{13}C NMR spectra of compound **RuL3** in CD_3CN.

3. High perfomance liquid chromatography and mass spectrometry

Table S1. HPLC method for complexes characterization.

Time (min)	Water (0.1% formic acid)	ACN (0.1% formic acid)
0-2	80	20
24-25	0	100
26-30	80	20

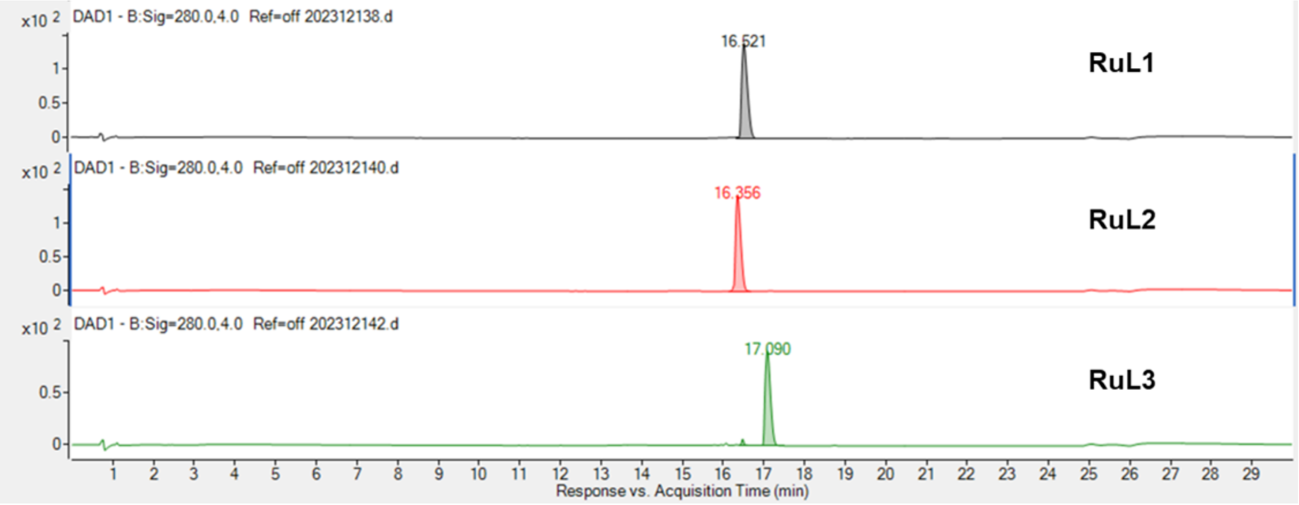

Figure S9. Reversed-phase HPLC traces of Ru(II) complexes with UV detection at 280 nm.

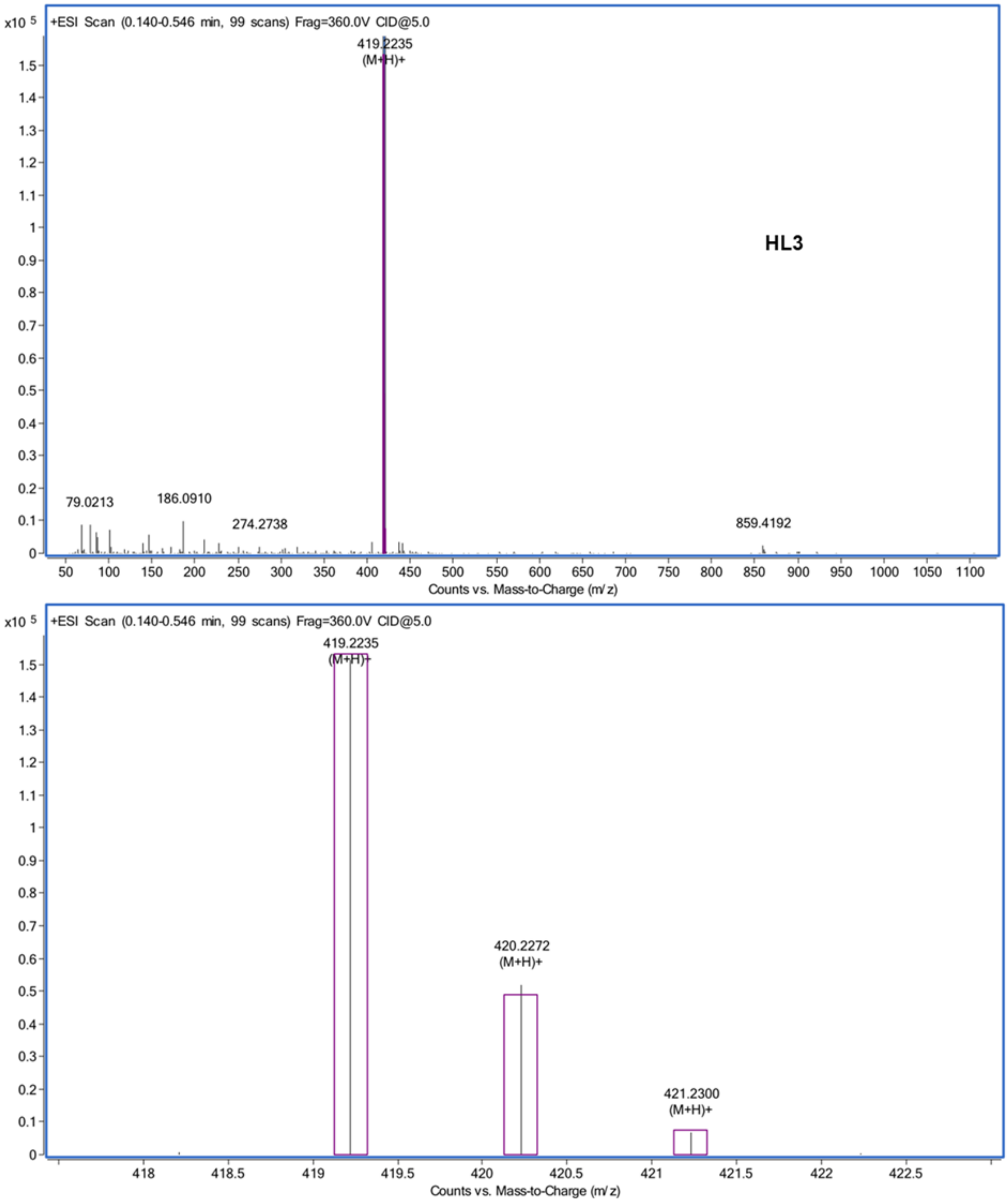

Figure S10. ESI-MS spectrum of **HL3** (positive detection mode).

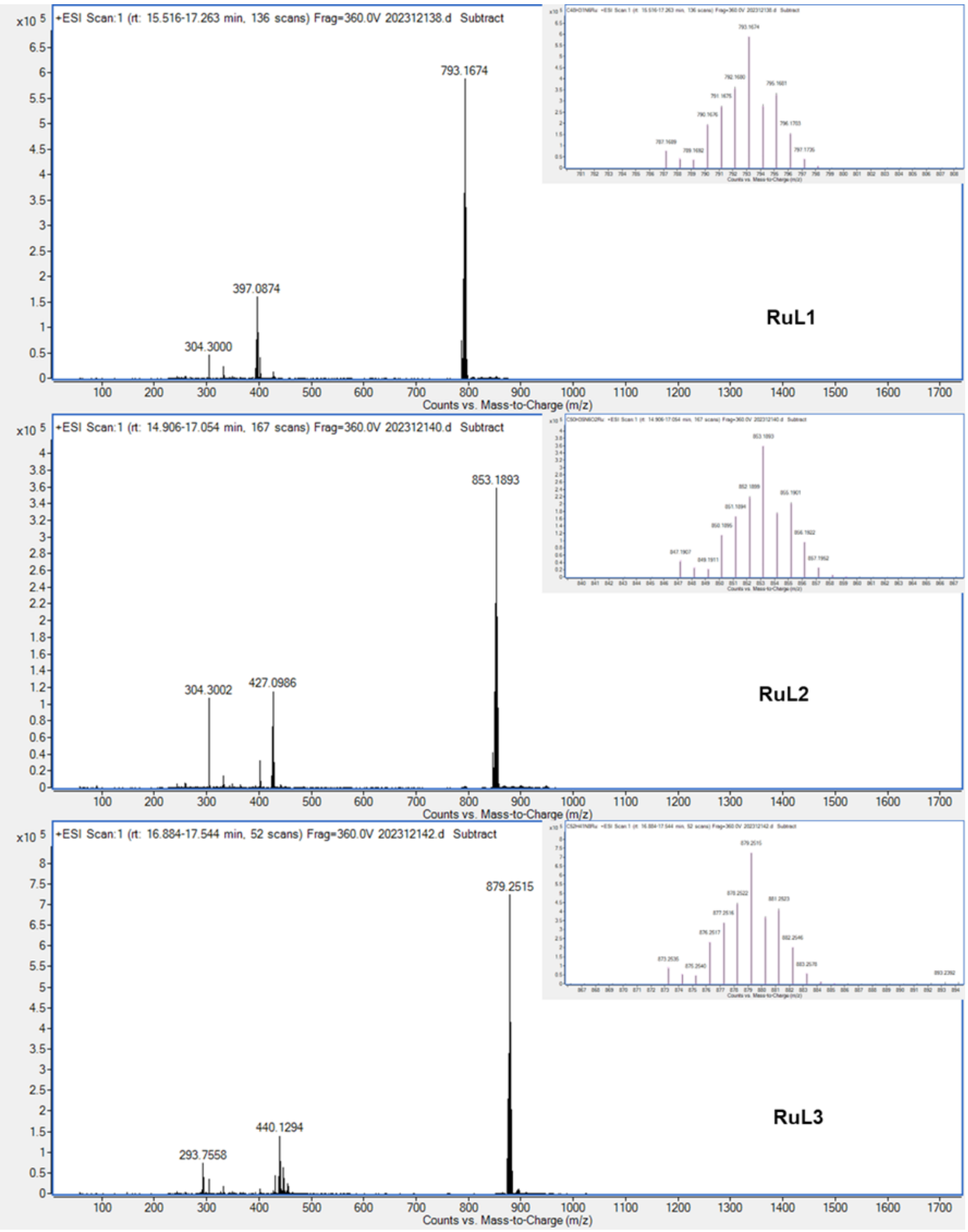

Figure S11. Mass spectra of the ~16 min peak of chromatograms of Figure S9 with peak of interest extracted for complexes **RuL1**-**RuL3**.

4. X-Ray crystallographic analysis for RuL1

Table S2. Crystal data and structure refinement for **RuL1**.

Empirical formula	C24.50 H16.50 Cl F3 N3 P0.50 Ru0.50
Formula weight	511.38
Temperature	100(2) K
Wavelength	0.71073 Å
Crystal system	Triclinic

Space group	P-1	
Unit cell dimensions	a = 12.3082(18) Å	a= 82.486(5)°.
b = 12.9969(19) Å	b= 71.564(6)°.	
c = 15.014(2) Å	g = 65.822(5)°.	
Volume	2078.7(5) Å^3	
Z	4	
Density (calculated)	1.634 Mg/m^3	
Absorption coefficient	0.617 mm^{-1}	
F(000)	1032	
Crystal size	0.160 x 0.140 x 0.010 mm3	
Theta range for data collection	1.895 to 28.325°.	
Index ranges	-16<=h<=16, -17<=k<=17, -20<=l<=20	
Reflections collected	98901	
Independent reflections	10365 [R(int) = 0.0406]	
Completeness to theta = 25.242°	100.0 %	
Absorption correction	Semi-empirical from equivalents	
Max. and min. transmission	0.7457 and 0.6557	
Refinement method	Full-matrix least-squares on F^2	
Data / restraints / parameters	10365 / 50 / 614	
Goodness-of-fit on F^2	1.113	
Final R indices [I>2sigma(I)]	R1 = 0.0414, wR2 = 0.0888	
R indices (all data)	R1 = 0.0535, wR2 = 0.0997	
Largest diff. peak and hole	1.853 and -0.900 e.Å^{-3}	

Table S3. Hydrogen bonds for **RuL1** a [Å and °].

D-H...A	d(D-H)	d(H...A)	d(D...A)	<(DHA)
C(2)-H(2)...F(1)#1	0.95	2.55	3.460(3)	161.5

Symmetry transformations used to generate equivalent atoms:
#1 x+1,y-1,z

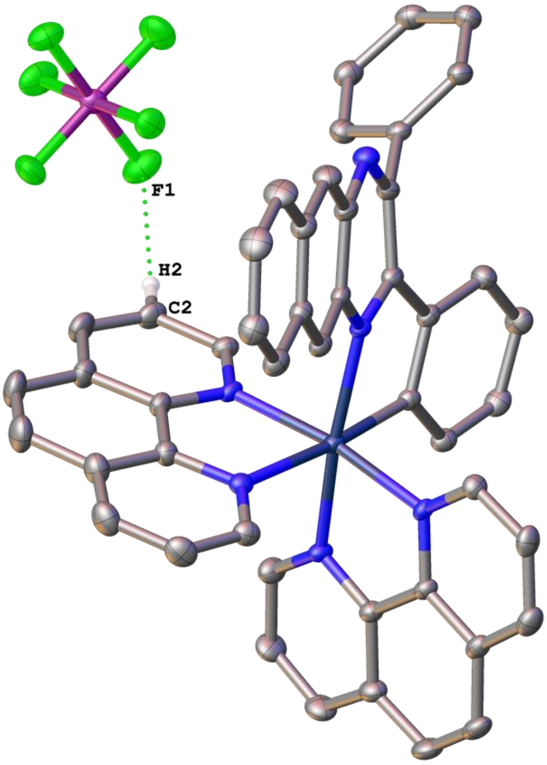

Figure S12. Intermolecular hydrogen bond interactions in the crystal structure of **RuL1**.

Table S4. π-π interaction parameters for **RuL1**.

	Cg-Cg distance [Å]	Dihedral angle[°]	Shift
Plane C4, C5, C6, C7, C8 and C9 with plane C4, C5, C6, C7, C8 and C9	3.725(2)	0.03(17)	1.511
Plane N4, C18, C21, C22, C23 and C24 with plane N4, C18, C21, C22, C23 and C24	3.5458(16)	0.03(13)	1.267

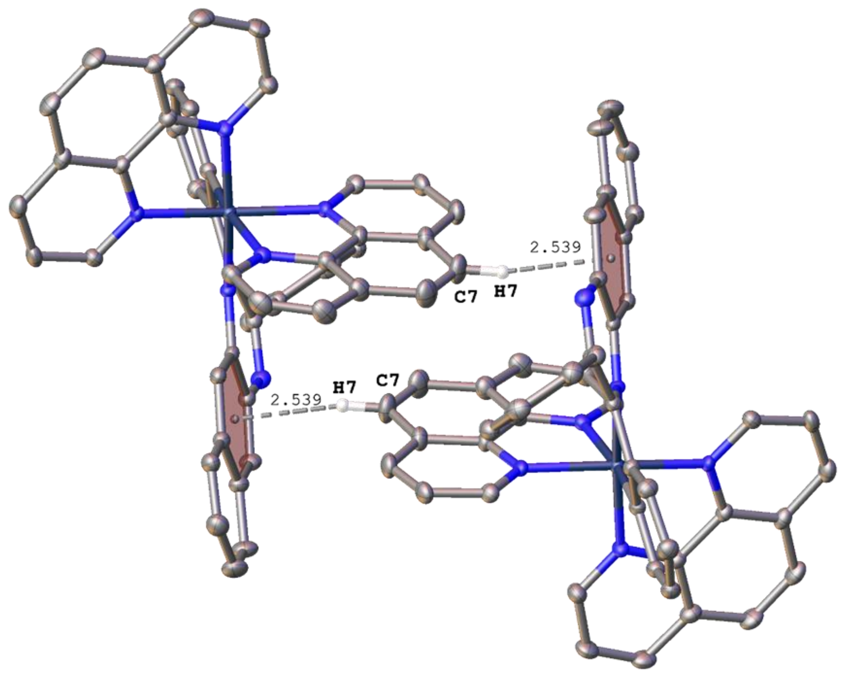

Figure S13. C-H···π interactions in the packing of **RuL1** in the crystal.

Table S5. C-H···π interaction parameters for **RuL1**.

	C7-centroid distance [Å]	H-Cg [Å]	Perpendicular distance from H to the plane of the ring	C-H···Cg angle [º]	Angle between vector Cg-H and the ring normal	Angle between the X-H bond and the pi plane
C7-H7···Cg1	3.411(3)	2.539	2.50	153	10.11	72

Cg1= atoms C25, C26, C27, C32, C33 and C34

5. Photophysical properties

Table S6. Absorption wavelengths and molar extinction coefficient of absorption spectra of the complexes **RuL1**-**RuL3** in acetonitrile and water (1% DMSO).

**Complex**	**λ [nm] (Ɛ [$M^{-1}cm^{-1}$])**
Acetonitrile	
**RuL1**	230 (84710), 265 (84930), h290 (44120), h320 (23110), 368 (11510), 410 (11690), 500 (10530), 570 (12600), 700 (1140)
**RuL2**	225 (83660), 265 (96010), h290 (46840), 325 (21230), 435 (18370), 560 (14300), 700 (1270)
**RuL3**	225 (88970), 265 (92160), h308 (41990), 383 (18020), 410 (18680), 505 (27020), h570 (14830), 700 (2330)
Water (1% DMSO)	
**RuL1**	270 (61910), h330 (24580), 415 (13310), 508 (9760), 590 (10760), 700 (1490)

**RuL2**	270 (74790), h294 (46370), 340 (19570), 445 (17610), 570 (11930), 700 (1630)
**RuL3**	270 (75870), h320 (33200), 420 (17010), 517 (21770), h600 (11260), 700 (3360)

6. Stability and photostability

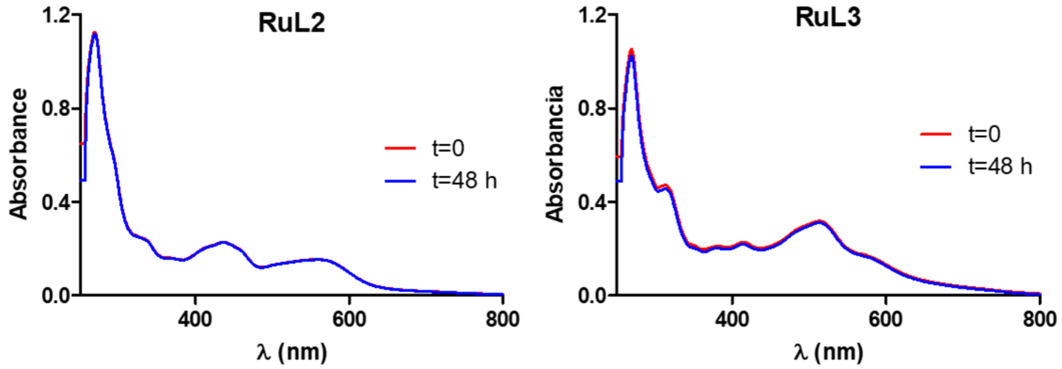

Figure S14. Time evolution of the absorption spectra of complexes **RuL2** and **RuL3** (10 µM) in DMSO at 37 °C.

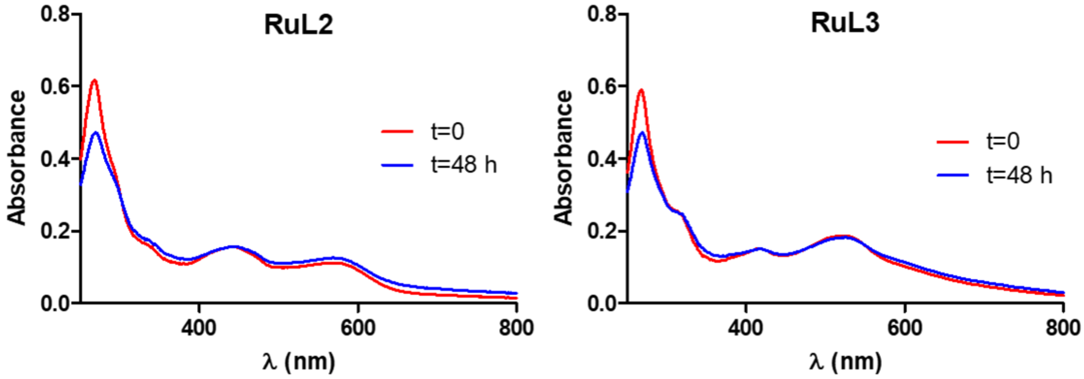

Figure S15. Time evolution of the absorption spectra of complexes **RuL2** and **RuL3** (10 µM) in RPMI (5% DMSO) at 37 °C.

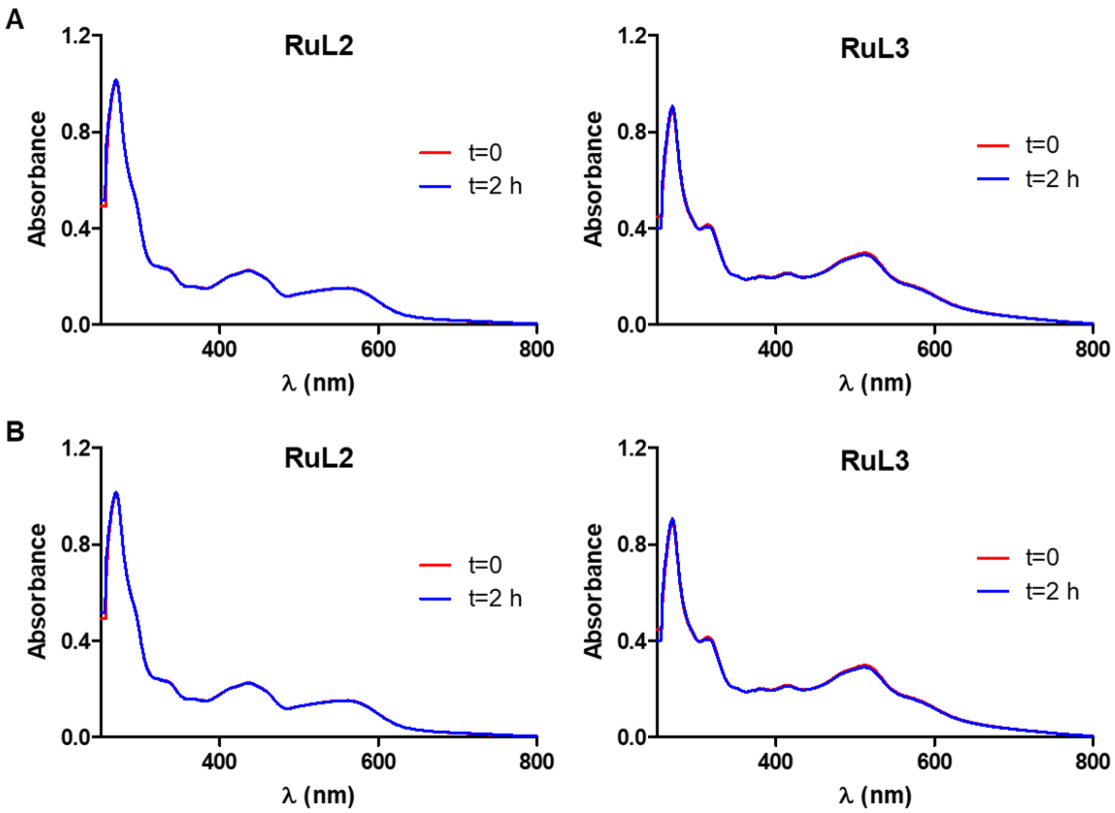

Figure S16. Time evolution of the absorption spectra of complexes **RuL2** and **RuL3** (10 µM) in DMSO under irradiation with (A) blue light (465 nm, 5 $mWcm^{-2}$), and (B) red light (620 nm, 15 mW/cm^2) for 2 h.

7. Evaluation of $^1O_2$ photogeneration

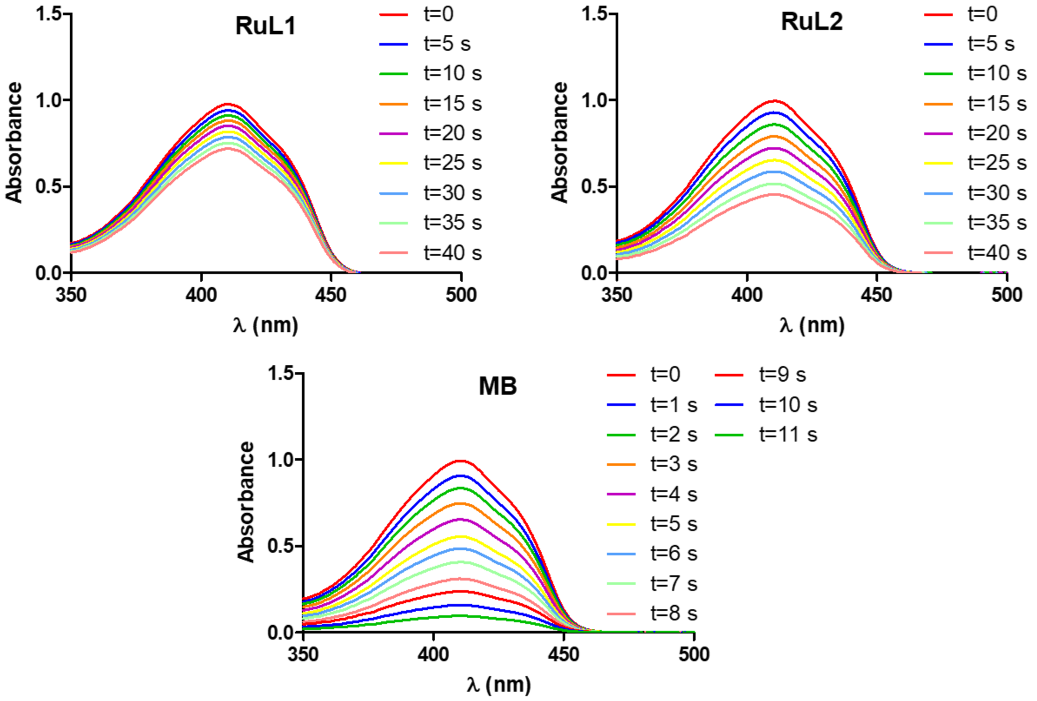

Figure S17. Decrease in the absorption intensity of DPBF in the presence of **RuL1**, **RuL2** or methylene blue after irradiation with red light (620 nm, 5.06 mW/cm²) in acetonitrile.

Table S7. Calculated values of ΦΔ for complexes **RuL1**-**RuL3** under red light irradiation (620 nm, 5.06 mW/cm²) in acetonitrile.

Complex	Φ_Δ[a]
**RuL1**	0.05
**RuL2**	0.09
**RuL3**	0.17

[a]Values of $\Phi_{\Delta,ref}$ for the reference used (methylene blue): 0.60.

8. Biological experiments

Table S8. IC_{50} values (μM)[a] obtained for cancer cells treated with the investigated Ru complexes in the dark or after irradiation as determined by the SRB assay.

	HCT116		
	Dark	Blue	Red
**RuL1**	0.93 ± 0.06	0.050 ± 0.009	0.12 ± 0.02
**RuL2**	0.70 ± 0.04	0.009 ± 0.002	0.09 ± 0.01
**RuL3**	0.88 ± 0.08	0.013 ± 0.003	0.064 ± 0.009

[a]Data represent mean ± SD from 3 independent experiments, each performed in triplicate.

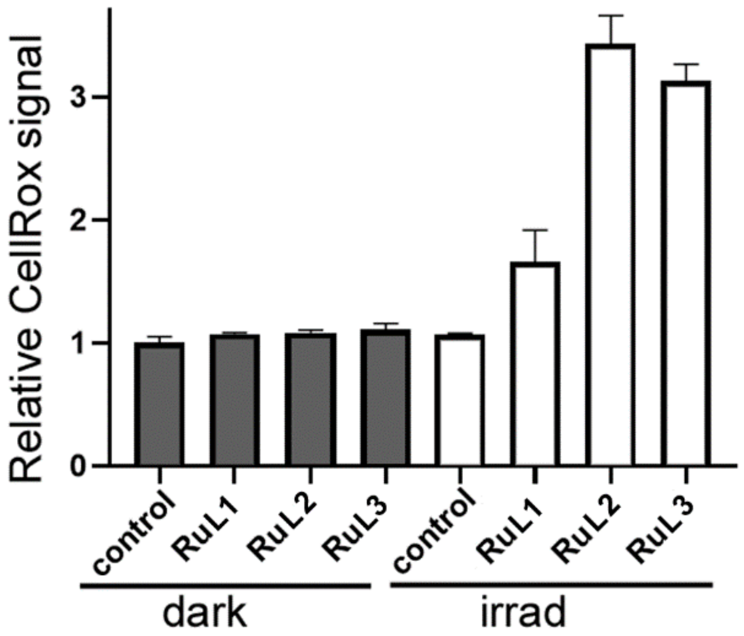

Figure S18. The efficiency of **RuL1**-**RuL3** (0.045 µM) to generate ROS in HCT116 cells in the dark or under irradiation with blue light. The fluorescence intensity of the control, untreated cells kept in dark was set as 1. The data represent a mean ± SEM of two independent experiments; in each experiment, at least 3 x10^4 cells were analyzed in individual samples, and the median of the fluorescence signal of the whole population was taken into evaluation.

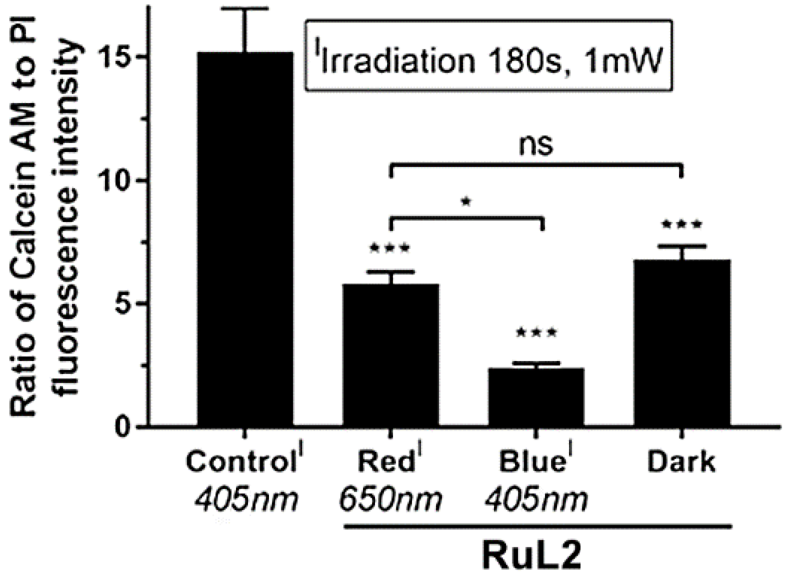

Figure S19: Ratio of Calcein AM to PI fluorescence intensity analyzed from the confocal microscopy microphotographs. Data were subjected to statistical analysis using Student's T-test with the following significance: *p≤0.05; ***p≤0.001; ns = nonsignificant. Data are the means from two independent experiments ±SDs.

9. Reference

[1] Phillips, K. A.; Stonelake, T. M.; Horton, P. N.; Coles, S. J.; Hallett, A. J.; O'Kell, S. P.; Beames, J. M.; Pope, S. J. A. *J. Organomet. Chem.* **2019**, *893*, 11–20.
